# Hepatic Global Transcriptomic Profiles of Holstein Cows According to Parity Reveal Age-Related Changes in Early Lactation

**DOI:** 10.3390/ijms24129906

**Published:** 2023-06-08

**Authors:** Zhangrui Cheng, Conrad Ferris, Mark A. Crowe, Klaus L. Ingvartsen, Clément Grelet, Amélie Vanlierde, Leslie Foldager, Frank Becker, D. Claire Wathes, the GplusE Consortium

**Affiliations:** 1 Department of Pathobiology and Population Sciences, Royal Veterinary College, Hawkshead Lane, North Mymms, Hatfield, Herts AL9 7TA, UK; dcwathes@rvc.ac.uk; 2 Agri-Food and Biosciences Institute, Newforge Lane, Upper Malone Road, Belfast BT9 5PX, UK; Conrad.Ferris@afbini.gov.uk; 3 School of Veterinary Medicine, University College Dublin, Belfield, D04 V1W8 Dublin, Ireland; mark.crowe@ucd.ie; 4 Department of Animal and Veterinary Sciences, Aarhus University, Blichers Allé 20, 8830 Tjele, Denmark; kli@anivet.au.dk (K.L.I.); leslie@anivet.au.dk (L.F.); 5 Valorisation of Agricultural Products Department, Walloon Agricultural Research Centre, 5030 Gembloux, Belgium; c.grelet@cra.wallonie.be (C.G.); a.vanlierde@cra.wallonie.be (A.V.); 6 Bioinformatics Research Centre, Aarhus University, Universitetsbyen 81, 8000 Aarhus, Denmark; 7 Research Institute for Farm Animal Biology, Wilhelm-Stahl-Allee 2, 18196 Dummerstorf, Germany; becker@fbn-dummerstorf.de

**Keywords:** liver, ageing, cellular senescence, transcriptome, immunity, metabolism, lactation, age, cows

## Abstract

Cows can live for over 20 years, but their productive lifespan averages only around 3 years after first calving. Liver dysfunction can reduce lifespan by increasing the risk of metabolic and infectious disease. This study investigated the changes in hepatic global transcriptomic profiles in early lactation Holstein cows in different lactations. Cows from five herds were grouped as primiparous (lactation number 1, PP, 534.7 ± 6.9 kg, *n* = 41), or multiparous with lactation numbers 2–3 (MP2–3, 634.5 ± 7.5 kg, *n* = 87) or 4–7 (MP4–7, 686.6 ± 11.4 kg, *n* = 40). Liver biopsies were collected at around 14 days after calving for RNA sequencing. Blood metabolites and milk yields were measured, and energy balance was calculated. There were extensive differences in hepatic gene expression between MP and PP cows, with 568 differentially expressed genes (DEGs) between MP2–3 and PP cows, and 719 DEGs between MP4–7 and PP cows, with downregulated DEGs predominating in MP cows. The differences between the two age groups of MP cows were moderate (82 DEGs). The gene expression differences suggested that MP cows had reduced immune functions compared with the PP cows. MP cows had increased gluconeogenesis but also evidence of impaired liver functionality. The MP cows had dysregulated protein synthesis and glycerophospholipid metabolism, and impaired genome and RNA stability and nutrient transport (22 differentially expressed solute carrier transporters). The genes associated with cell cycle arrest, apoptosis, and the production of antimicrobial peptides were upregulated. More surprisingly, evidence of hepatic inflammation leading to fibrosis was present in the primiparous cows as they started their first lactation. This study has therefore shown that the ageing process in the livers of dairy cows is accelerated by successive lactations and increasing milk yields. This was associated with evidence of metabolic and immune disorders together with hepatic dysfunction. These problems are likely to increase involuntary culling, thus reducing the average longevity in dairy herds.

## 1. Introduction

The profitability of dairy enterprises increases with greater cow longevity, associated with a higher proportion of total lifetime spent in milk production [1]. To optimise economic performance, heifers are expected to have their first calf at 24 months of age and continue to calve at annual intervals until their fourth or fifth lactation [2]. However, genetic selection in favour of high milk yields has led to the decreased fertility and lifespan of Holstein dairy cows [3]. While cattle can live for over 20 years, in practice their lifespan is currently around 4.5–7 years, representing 2.5–5 lactations [1,4,5]. Around 25% of dairy cows are culled in each lactation [6], with only around 40% of the herd surviving beyond their third lactation. This is economically inefficient as it results in the loss of milk production and requires keeping more animals for the same milk output. In addition, short lifespans result in more methane emissions per cow, as their productive life after first calving is proportionately less in relation to their total lifespan, thus having a negative environmental impact [7]. The main causes of culling are poor fertility and milk production, mastitis, and lameness [8,9].

During the transition period, dairy cows undergo pregnancy, delivery, and lactogenesis, experience many endocrine changes that are associated with regulating these events, and are prone to oxidative stress [10]. After calving, the increased energy demand for lactation necessitates extensive alterations in metabolism and energy supply [11,12]. Insufficient feed intake or the excessive mobilisation of body lipids can lead to a period of postpartum negative energy balance (NEB) [13,14]. Homeorhetic regulation involving the liver is central in dealing with the increased nutrient need in early lactation. This includes increased lipolysis and downregulating triacylglycerol synthesis in adipose tissue, and upregulating fatty acid oxidation and gluconeogenesis [14,15,16]. This results in changes in circulating metabolites, including increased concentrations of non-esterified fatty acids (NEFA) and β-hydroxybutyrate (BHB), and decreased glucose concentrations, all of which contribute to the reduced immune capacity at this time [17,18]. The liver also plays a co-ordinating role in cholesterol homeostasis [19]. Excessive tissue mobilisation may lead to the development of hepatic steatosis, which compromises glucose production and increases inflammatory responses [10]. NEB is an important contributor to immunosuppression after calving [20]. Previous studies have shown that the cows with more severe postpartum NEB had reduced milk yield and fertility [21], prolonged uterine remodelling and repair [22], and impaired local and systemic immunity [23].

In addition to food digestion, detoxification, and metabolism, the liver synthesises and secretes many inflammatory mediators [24]. The hepatic production of acute phase proteins (APPs) is altered in response to inflammatory cytokines (such as IL1, IL6, and TNFA), contributing to innate immune processes against invading microorganisms [25,26]. The liver is also the main source of insulin-like growth factor-1 (IGF-1) and its binding proteins (IGFBPs) [27]. These are important for cellular metabolism and proliferation, and also play roles in both innate and acquired immunity [28]. Furthermore, the liver interacts with the reproductive system in a multifaceted fashion, and can modulate the metabolism and transport of steroids to tissues via the altered secretion of sex hormone-binding globulin [29].

The hallmarks of ageing in the liver, demonstrated in both human populations and a variety of model organisms, include genome instability, telomere attrition, epigenome alteration, loss of proteostasis, response to endoplasmic reticulum stress, deregulated nutrient sensing, mitochondrial dysfunction, cellular senescence, stem cell exhaustion, and altered intercellular communication [30,31,32]. These changes cause a progressive impairment in the ability of an organism to maintain homeostasis and are associated with increased incidences of both hepatic and systemic diseases [33]. We previously compared circulating leucocyte global transcriptomic profiles between cows with different lactation numbers, finding changes in many genes and pathways that were comparable to those known to be associated with ageing in humans and model organisms. In addition, alterations in energy utilisation and immune response in the leucocytes of older cows were observed [34].

There is currently a lack of information regarding hepatic changes during the ageing process in dairy cows. In the present study the hepatic transcriptomic gene expression profiles in early lactation between young (lactation number 1), medium (lactations 2–3), and older cows (lactations 4–7) were compared using next generation sequencing and bioinformatics approaches. This approach sheds light on the changes in genes and pathways involved in age-related symptoms and diseases arising during the different phases of a cow’s life. Our hypothesis was that this would show a decreasing ability of the older animals to cope with the postpartum changes in metabolism and immunity, thus acting as an important determinant of their lifespan.

## 2. Results

### 2.1. Blood Metabolites and Animal Performance Traits between the Three Lactation Groups

Table 1 shows the results of blood metabolites, feed intake, milk yield and composition, body weight (BW), body condition score (BCS), energy corrected milk (ECM), and energy balance (EBAL) between the three lactation groups at around 14 days in milk (DIM). Circulating glucose and IGF-1 concentrations were both significantly reduced as the lactation number increased (PP > MP2–3 > MP4–7, *p* < 0.01). Circulating NEFA concentrations were higher in MP4–7 than in PP cows (*p* < 0.01) or MP2–3 (*p* < 0.05) but did not differ significantly between PP and MP2–3 cows. There was also a trend (*p* = 0.07) for increased concentrations of BHB with age. Both MP groups had significantly higher cholesterol concentrations (*p* < 0.05) and daily dry matter intakes (DMI) (*p* < 0.0001) than the PP cows. BW, milk yield (MY), and ECM all increased with lactation number (MP4–7 > MP2–3 > PP, *p* < 0.01), whereas the BCS was greater at day 14 in the PP cows than in either MP group (*p* < 0.05). MP4–7 cows had a more negative EBAL than the younger cows (*p* < 0.01), whereas the difference between the PP and MP2–3 groups was not significant. The overall incidence of health conditions diagnosed in the first 14 days in milk (i.e., preceding hepatic biopsy collection) increased from 9.7% in PP cows to 13.8% in MP2–3 cows and 32.5% in MP4–7 cows (Supplementary file Table S1A).

### 2.2. Hepatic Gene Expression Profiles between the Three Lactation Groups

**Figure 1:**
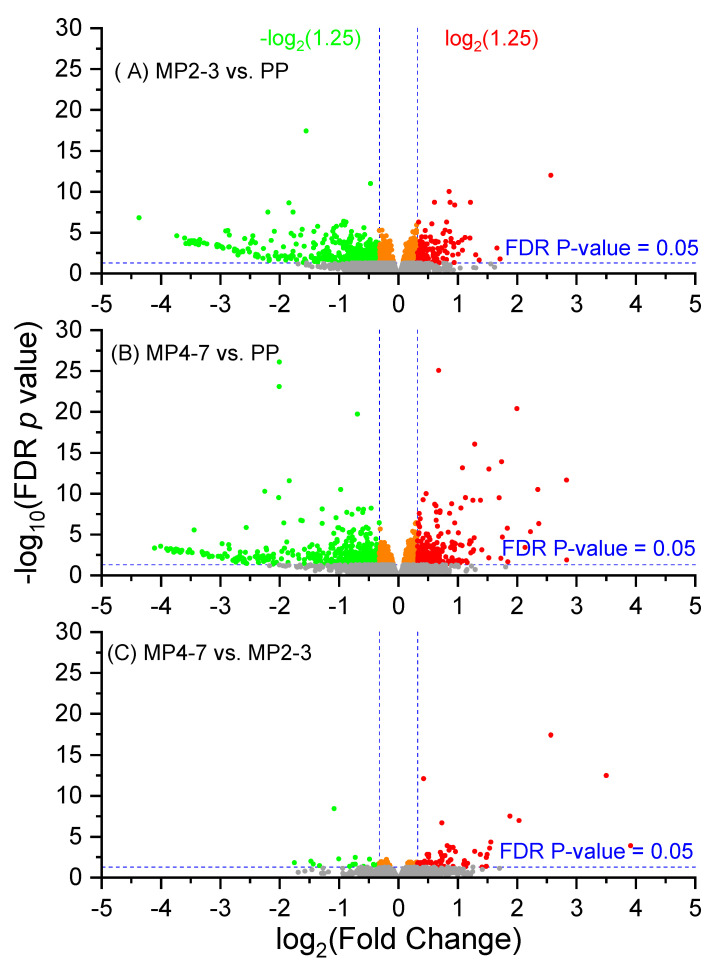
Volcano plots showing the gene expression profiles (**A**) between the MP2–3 (*n* = 87) and PP (*n* = 41) cows; (**B**) between the MP4–7 (*n* = 40) and PP (*n* = 41) cows; and (**C**) between the MP 4–7 (*n* = 40) and MP2–3 (*n* = 41) cows. The reads were quantified as Reads Per Kilobase Million (RPKM). The fold changes were log_2_-transformed, and the *p*-values were BH-adjusted to control the FDR at level 0.05. The green dots indicate the downregulated genes with FDR (BH) *p* < 0.05 and fold changes ≤ −log_2_ (1.25), and red dots indicate upregulated genes with FDR (BH) *p* < 0.05 and fold changes ≥ log_2_ (1.25). The orange dots indicate the genes with *FDR* (BH) *p* < 0.05 but with absolute fold changes < log_2_ (1.25).

**Figure 2:**
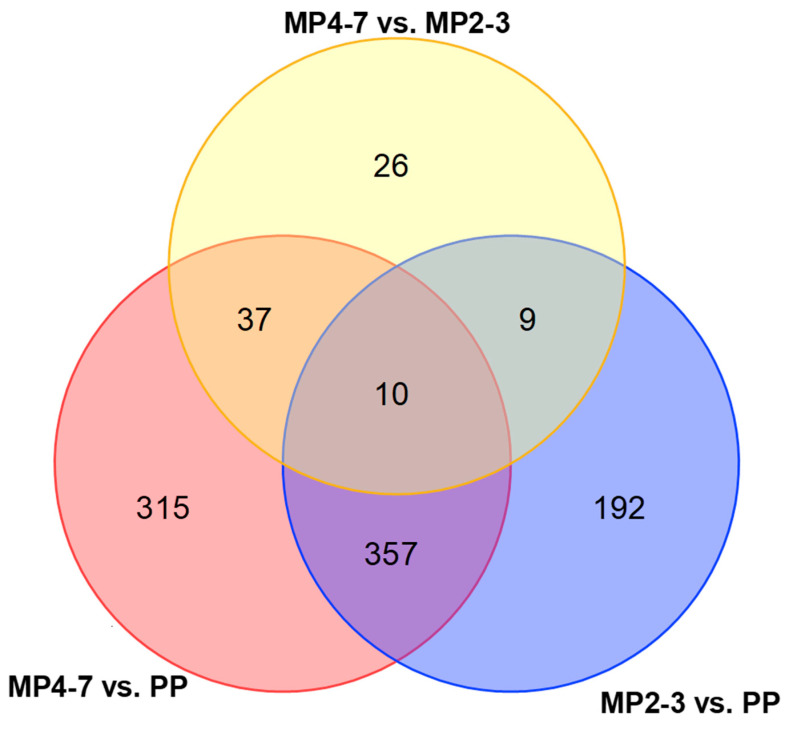
Venn diagram showing the numbers of hepatic differentially expressed genes between the three age groups.

The reference genome of *Bos taurus* ARS-UCD 1.2 contains 35,158 genes, of which 19,591 were mapped and quantifiable in the samples, with the maximum group value of 29,255 RPKM for *ALB*. Volcano plots showing the differential gene expression between the three lactation groups are given in Figure 1. A Venn diagram showing the DEGs derived from the three comparisons is presented in Figure 2. While there were only 10 common DEGs (Supplementary file Table S1B) between all three comparisons, the DEGs derived from the comparisons of MP4–7 with the PP group included most of the DEGs generated in the three comparisons.

### 2.3. Comparison of Hepatic Gene Expression between MP Cows with 4–7 Lactations and the PP Cows

The comparisons of hepatic gene expression between the MP4–7 and PP groups generated 719 DEGs, with 260 being upregulated and 459 downregulated (Supplementary file Table S2A). The top 20 upregulated DEGs ranked by FDR (BH) *p*-values, which were expressed at higher levels in the older cows, are given in Supplementary file Table S2B. These included six DEGs associated with protein metabolism (*CCNB3*, *FBXW5*, *RARRES1)* and other metabolic processes (*BARX2*, *DUSP3*, *MYOM1)* and five involved in response to a stimulus (*DUSP3*, *HSPA6*, *MYOM1*, *RASL11B*, *TENM1*). *GRAMD4* encodes a protein involved in cell cycle control and apoptosis. Several DEGs are involved in various binding activities, such as a calcium ion binding/sensor (*NCS1*), protein binding (*KLHDC7A*, *SLC13A2*), and DNA and histone binding (*SPTY2D1*).

The top 20 downregulated DEGs, with reduced expression in older cows, are given in Supplementary file Table S2C. Half of these are involved in metabolic processes including protein metabolism (*ADAMTS12*, *AGTR2*, *COL1A1*, *COL3A1*, *COL4A5*, *DNAJC18*, *NBDY*) and other metabolic processes *(IGF1*, *LRP3*, *MCOLN2*, *NFATC4*). Five DEGs are associated with the developmental process (*COL1A1*, *COL3A1*, *LRP3*, *RTN4RL1*, *SPARC*) and nine are involved in the response to a stimulus (*ADAMTS12*, *CCDC80*, *COL1A1*, *COL3A1*, *COL4A5*, *IGF1*, *NFATC4*, *RTN4RL1*, *S100A16*). Some of these genes encode proteins involved in multiple biological processes. For example, *IGF1* is associated with cellular proliferation, response to stimulus and nutrient levels, and glucose metabolism. *COL1A1* and *COL3A1* play roles in metabolism, adhesion, locomotion, multicellular processes, response to stimuli, development, and protein metabolism. *IGF2BP3* binds to nucleic acid, promotes cell adhesion, and is known to be involved in RNA synthesis and metabolism. *RTL4* may have a role in inflammation while *GSTP1*_2 belongs to a family of enzymes that play an important role in detoxification.

**Figure 3:**
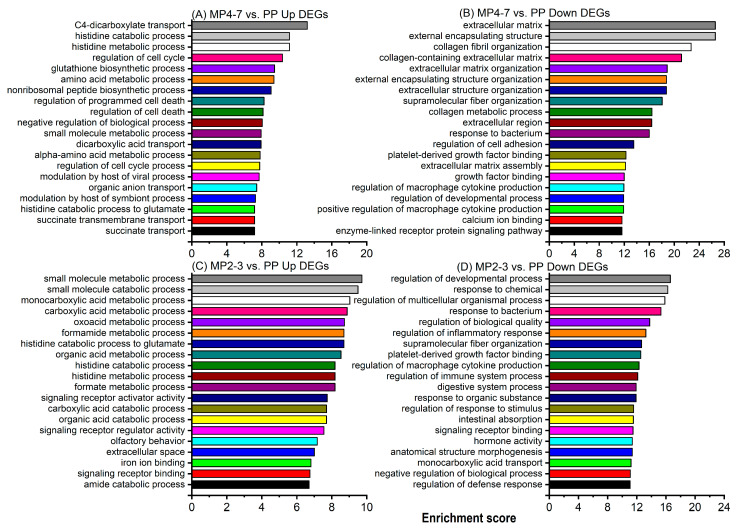
Top 20 GO functions associated with (**A**) upregulated DEGs between MP4–7 and PP cows, (**B**) downregulated DEGs between MP4–7 and PP cows, (**C**) upregulated DEGs between MP2–3 and PP cows, and (**D**) downregulated DEGs between MP2–3 and PP cows.

GO enrichment analysis was carried out to identify the biological functions involved. The upregulated DEGs (260) between MP4–7 and PP cows were significantly enriched with 480 GO functions. The top 20 functions (Figure 3A) are associated with metabolism, the transport of various chemicals, cell cycle control, and the maintenance of cellular homeostasis. The processes ‘regulation of programmed cell death’ and ‘regulation of cell death’ had an enrichment score (ES) > 8 and contained 25 upregulated DEGs (*ANGPTL4*, *ARG2*, *ATF3*, *BIRC5*, *CCL5*, *CCND1*, *CDK1*, *CLCF1*, *DDIAS*, *DYRK3*, *ECT2*, *GPNMB*, *LTF*, *MAD2L1*, *MAGED1*, *MECOM*, *NOD1*, *PDK4*, *PRF1*, *PRLR*, *RPS6KA2*, *SIK1*, *SOX9*, *TGFB2*, *ZBTB16*).

**Figure 4:**
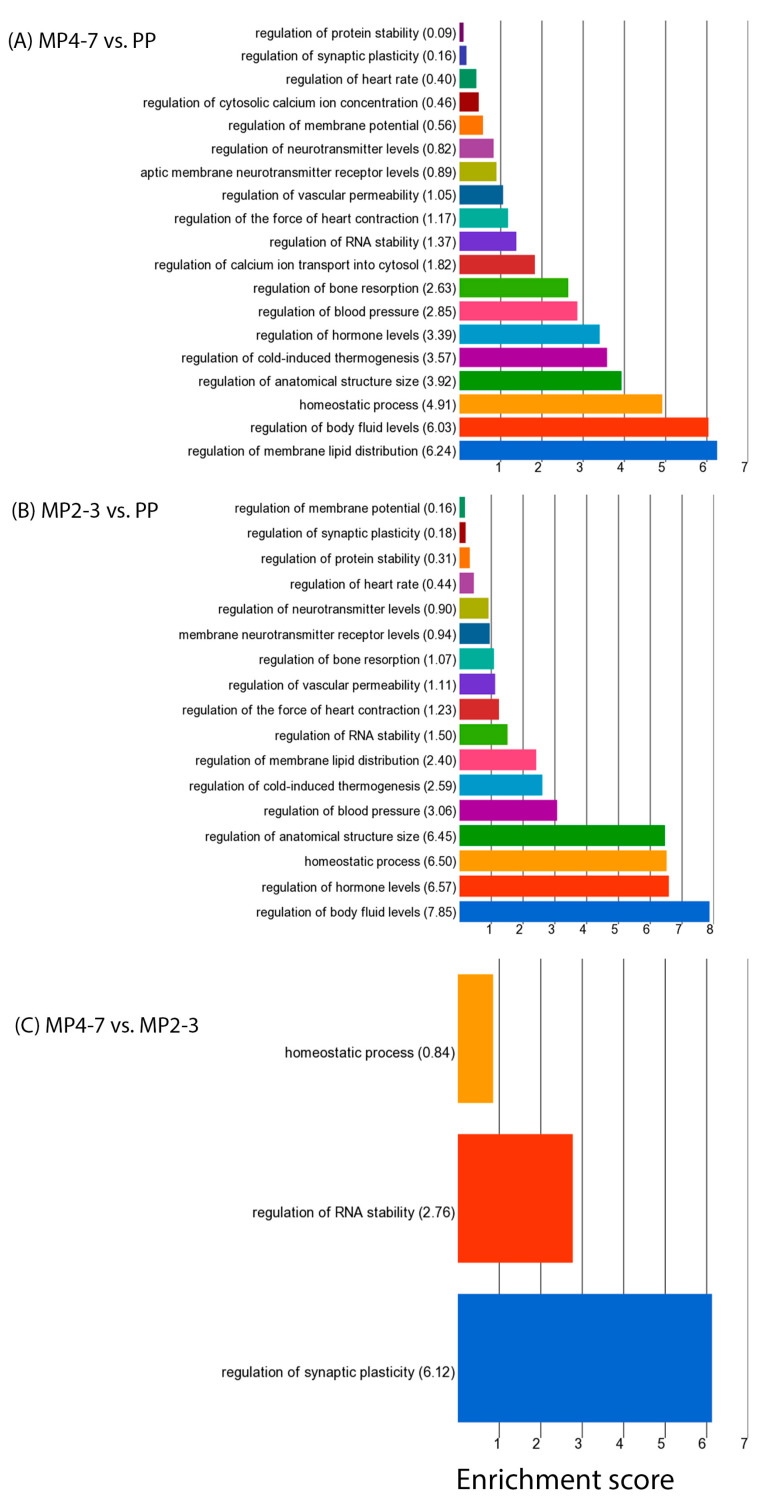
Gene Ontology biological process of regulation of biological quality associated with the downregulated DEGs derived from (**A**) comparison of MP4–7 (*n* = 40) with PP (*n* = 41) cows, (**B**) MP2–3 (*n* = 87) with PP cows, and (**C**) MP4–7 with MP2–3 cows.

When the 459 downregulated DEGs were used for GO enrichment analysis, 751 functions were significantly enriched, with the top 20 presented in Figure 3B. Among them, many were associated with the extracellular matrix, such as external encapsulating structure (22 DEGs), extracellular matrix (22 DEGs), collagen-containing extracellular matrix (16 DEGs), etc. (Supplementary file Table S3A). Three biological processes (response to bacterium, regulation of macrophage cytokine production, and positive regulation of macrophage cytokine production) were associated with immunity. The GO biological process of ‘regulation of biological quality’ contained 65 downregulated DEGs, playing roles in the regulation of membrane lipid distribution, body fluid levels, the homeostatic process, anatomical structure size, thermogenesis, and hormone levels (Figure 4A and Supplementary file Table S3B).

**Figure 5:**
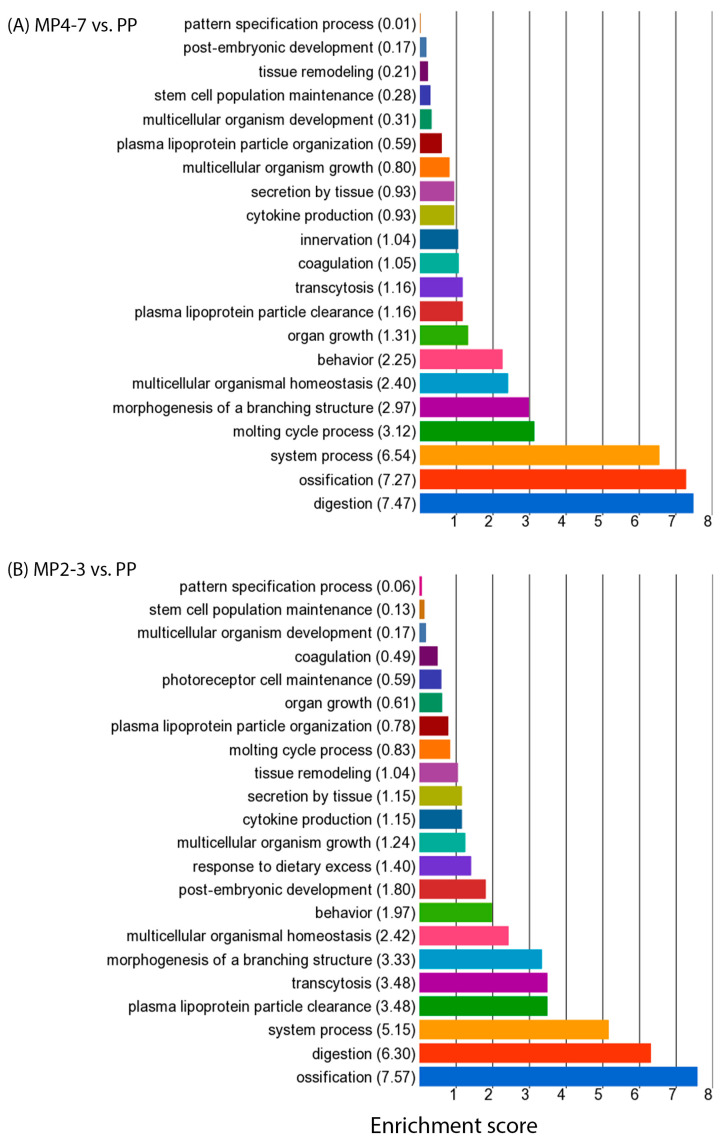
Gene Ontology biological process of multicellular organismal process associated with the DEGs derived from (**A**) comparison of MP4–7 (*n* = 40) with PP (*n* = 41) cows, and (**B**) MP2–3 (*n* = 87) with PP cows.

**Figure 6:**
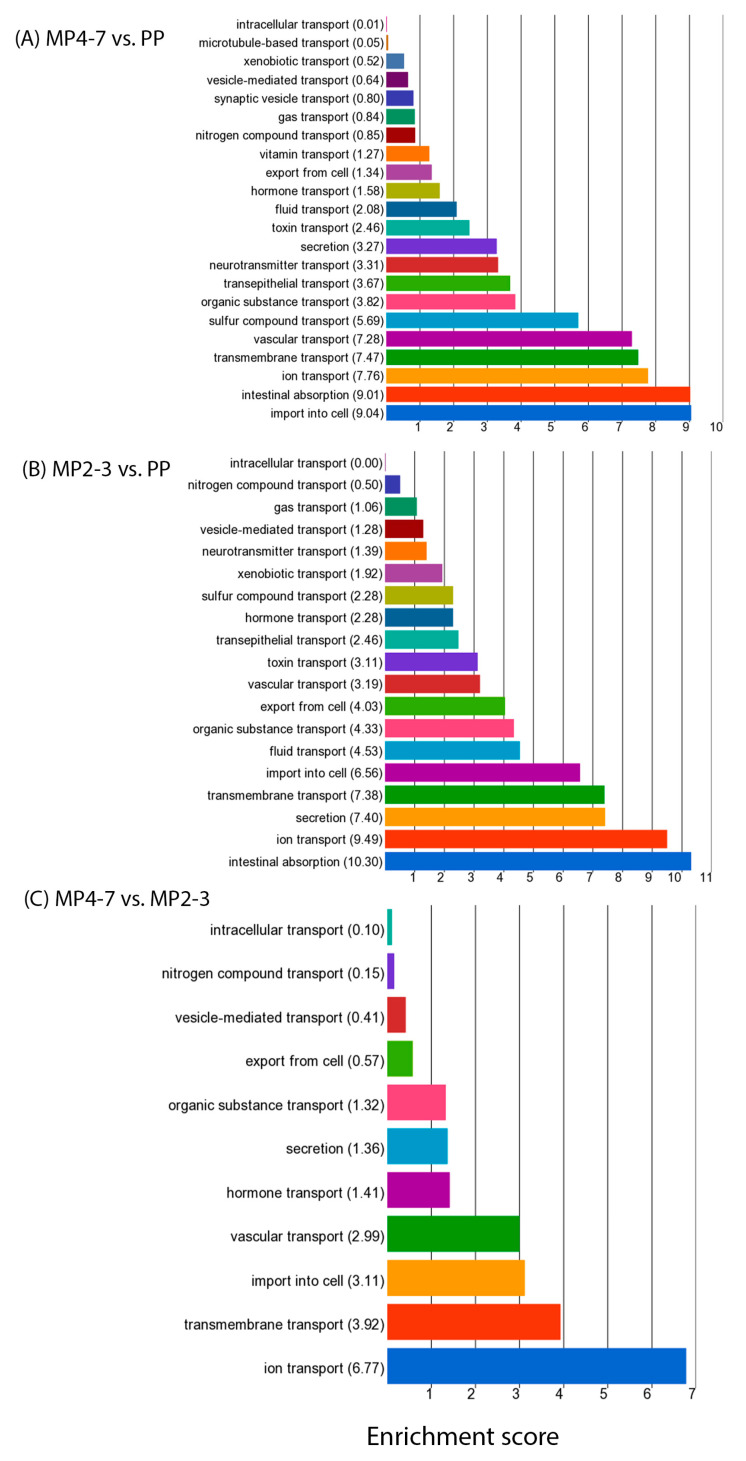
Gene Ontology biological process of transport associated with the DEGs derived from the comparison of (**A**) MP4–7 (*n* = 40) with PP (*n* = 41) cows, (**B**) MP2–3 (*n* = 87) with PP cows, and (**C**) MP4–7 with MP2–3 cows.

All functions associated with both up- and downregulated DEGs were then summarised with a GO browser. Seven categories of GO biological processes were significant, most of which were associated with a large number of DEGs, which are listed in the Supplementary files (See Table 2 for details). All categories were predominantly enriched with the genes that were downregulated in the MP4–7 cows. The interspecies interaction between organisms was on the top, with 39 DEGs mainly involved in the immune defence against microbial organisms. The multicellular organismal process was associated with 81 DEGs, with digestion on the top of the sub-biological functions (Figure 5A). The process of biological regulation contained 309 DEGs, playing roles in the regulation of biological quality, molecular function, and biological processes. The GO function ‘Localization’ includes any process in which a cell, a substance, or a cellular entity is transported, tethered to, or otherwise maintained in a specific location. The only significant sub-function was transport, which was associated with 68 downregulated and 29 upregulated DEGs playing roles in the uptake and transport of a wide range of molecules (Figure 6A and Supplementary file Table S3I). Two significant roles were identified within the function ‘developmental process’: anatomical structure morphogenesis and anatomical structure development.

KEGG pathway enrichment using all DEGs derived from the comparison of MP4–7 with PP cows identified 25 significantly altered pathways, of which 18 were predominantly enriched with downregulated DEGs. These were mainly involved in aspects of protein and lipid metabolism, and in various signalling pathways (Table 3). Eight pathways included immune and inflammatory processes (amoebiasis, prostate cancer, rheumatoid arthritis, relaxin, viral protein interaction with cytokine and cytokine receptor, MARK signalling, AMPK signalling, and PI3K-Akt signalling). Some of these pathways seemed unrelated to the liver or female animals (such as prostate cancer), but were associated with inflammatory processes and their related genes. In the older cows, there were 17 DEGs associated with protein digestion and absorption (*CELA2A*, *CELA3B*, *COL12A1*, *COL15A1*, *COL1A1*, *COL1A2*, *COL3A1*, *COL4A5*, *COL5A1*, *COL5A2*, *CPA1*, *CPB1*, *CTRB1*, *ELN*, *MEP1B*, *SLC15A1*, *SLC3A1*), six with fat digestion and absorption (*CEL*, *NPC1L1*, *PLA2G1B*, *PNLIP*, *PNLIPRP2*, *SLC27A4*), and six with vitamin digestion and absorption (*CUBN*, *FOLH1B*, *PLB1*, *PNLIP*, *RBP2*, *SLC46A1*). The other predominantly downregulated pathways included extracellular matrix–receptor interaction (down, *COL1A1*, *COL1A2*, *COL4A5*, *GP1BA*, *ITGB4*, *LAMA2*, *TNC*, *TNXB:* up *HMMR*) and the oestrogen signalling pathway (down, *CREB3L1*, *CREB5*, *GABBR2*, *GNAO1*, *KRT20*, *MMP2*, *PRKCD*, *SRC*, *TGFA:* up, *HSPA1A*, *HSPA2*, *HSPA6*).

### 2.4. Comparison of Hepatic Gene Expression between the MP Cows with 2–3 Lactations and the PP Cows

There were 568 DEGs in the comparison between MP2–3 and PP cows, with more downregulated than upregulated (433 vs. 135) (Supplementary file Table S4A). Among the top 20 upregulated DEGs (Supplementary file Table S4B), 8 were in common with the top 20 DEGs in the MP4–7 vs. PP comparison (*BARX2*, *COL27A1*, *DUSP3*, *KLHDC7A*, *MYOM1*, *NCS1*, *SLC13A2*, *TENM1*). For the other DEGs, *AMDHD1*, *CTSV*, *GPNMB*, *DUSP26*, *SP5*, and *TRHDE* are involved in metabolic processes. *MFSD2A* plays roles in several biological processes, including metabolism, the multicellular organismal process, response to stimulus, development, fatty acid and protein metabolism, and response to nutrient levels, while *IGFBP2* is involved in the regulation of growth and metabolism and *CTSV* in immune processes.

Among the top 20 downregulated DEGs (Supplementary file Table S4C), there were 7 in common with the top 20 generated from the comparison between MP4–7 and PP cows (*CCDC80*, *DNAJC18*, *IGF2BP3*, *GIPC2*, *LRP3*, *TRPC5*, *XK*). Other DEGs play roles in protein metabolism (*HOPX*, *INHBA*, *NOD2*, *TRIM31*), other aspects of metabolism (*AK4*, *JCHAIN*, *LRP3*, *MGC138914*, *TNC*), response to stimulus (*ADM2*, *AK4*, *CCDC80*, *HOPX*, *INHBA*, *JCHAIN*, *NOD2*, *TNC*), and the developmental process (*HOPX*, *IGF2BP3*, *INHBA*, *LRP3*, *TNC*). Several DEGs have multiple roles in biological processes. For example, *HOPX*, *INHBA*, *JCHAIN*, *NOD2*, and *TNC* play roles in the processes of immunity, metabolism, multicellular organism, and response to stimulus in which *JCHAIN* is also an antimicrobial gene. *DNAJC18* is involved in the cellular response to a misfolded protein, chaperone cofactor-dependent protein refolding, and the ubiquitin-dependent ERAD pathway (endoplasmic reticulum-associated degradation).

The GO enrichment analysis showed that the upregulated DEGs were significantly associated with 366 GO functions, with the top 20 given in Figure 3C. Fourteen of these were associated with the metabolic and catabolic processes of various molecules. A greater number of downregulated DEGs produced more significant functions (838), with higher enrichment scores and more DEGs associated with each function. The top 20 functions are illustrated in Figure 3D. The top function (ES = 17) was the ‘regulation of developmental process’ with 60 downregulated DEGs, which play roles in the regulation of anatomical structure morphogenesis, cell differentiation, and development. Five biological processes are involved in immune defence, of which ‘response to bacterium’ came fourth with ES = 15 and 17 associated DEGs (*ADIPOQ*, *CASP1*, *CCDC80*, *CHGA*, *GZMA*, *IL22RA1*, *JCHAIN*, *LCN2*, *LEAP2*, *LYPD8*, *NOD2*, *PLA2G1B*, *PNLIPRP2*, *PRKCD*, *PYCARD*, *REG4*, *VIL1*). These are involved in defence responses to Gram-negative bacteria (*CASP1*, *CHGA*, *IL22RA1*, *LYPD8*, *PYCARD*), Gram-positive bacteria (*CHGA*, *PLA2G1B*, *PYCARD*), and the antibacterial humoral response (*JCHAIN*, *PLA2G1B*). The biological process of regulation of macrophage cytokine production was enriched with six DEGs playing roles in both the positive (*CASP1*, *NOD2*, *PYCARD*, *WNT5A*) and negative (*IRAK3*, *TGFB3*) regulation of cytokine production. The GO function ‘regulation of biological quality’ with an ES = 14 contained 66 DEGs which play various regulatory roles in body fluid levels, hormone levels, homeostatic processes, anatomical structure size, and blood pressure (Figure 4B and Supplementary file Table S5A). Several functions were related to the maintenance of homeostasis, including the developmental process, multicellular organismal process, digestive system process, and intestinal absorption.

The GO browser summarised the functions derived from both up- and downregulated DEGs into nine significant GO categories (Table 4) which were associated with the maintenance of homeostasis (biomineralisation, multicellular organismal process, developmental process, biological regulation, and localisation) and immune defence (interspecies interaction between organisms, immune system process, locomotion, and response to stimulus). These were predominantly enriched with the downregulated DEGs. Transport was the only significant sub-GO function within ‘localisation’, with mainly downregulated DEGs playing significant roles in the transport of a wide variety of molecules (Figure 6B and Supplementary file Table S5J). ‘Multicellular organismal process’ contained a number of significant sub-functions, including ossification, digestion, system process, plasma lipoprotein particle clearance, transcytosis, and the morphogenesis of a branching structure (Figure 5B).

Fifteen significant pathways were identified by KEGG pathway analysis (Table 5). Among them, 13 were in common with the pathways derived from MP4–7 vs. PP comparisons (Table 3), but with smaller enrichment scores and fewer associated DEGs. Downregulated DEGs again predominated. One additional pathway was cytokine–cytokine receptor interaction, associated with 18 DEGs (down, *CCL1*, *CCL24*, *CCL25*, *CCL26*, *CCR9*, *CTF1*, *IL1RL1*, *IL22RA1*, *INHBA*, *INHBE*, *TGFB3*, *XCL2;* up, *CCR1*, *CD70*, *CLCF1*, *IL1R2*, *PRLR*, *TNFSF9*). The other was the metabolism of xenobiotics by cytochrome P450, which was enriched with one downregulated (*UGT1A6*) and five upregulated DEGs (*ADH4*, *CYP1A2*, *GSTM2*, *GSTT2*, *MGC127133*).

### 2.5. Comparison of Hepatic Gene Expression between the MP Cows with 4–7 and 2–3 Lactations

A comparison of the hepatic gene expression between the MP cows with 4–7 and 2–3 lactations generated 82 DEGs, of which 66 were expressed at a higher level in the older age group (Supplementary file Table S6A). The top 20 upregulated and 16 downregulated DEGs are listed in Supplementary files Table S6B,C. A number of both down- and upregulated DEGs were associated with immune defence (such as *HSPA6*, *TRAT1*, *PTX3*, *LOC618565*, *CDKN2A*, *BCL6*, *IFI16* in the upregulated list and *AGER*, *NFATC4*, *MCOLN2* in the downregulated list).

The GO enrichment analysis showed that the upregulated DEGs were associated with 347 GO functions, with the top 20 listed in Supplementary file Table S7A. Protein folding chaperone was on top (ES = 11) with four associated DEGs (*DNAJB1*, *HSPA1A*, *HSPA6*, *HSPH1*), all of which are associated with ATP-dependent protein folding. There was also a clear theme of the immune process, including responses to various stimuli and stressors. The biological processes of positive regulation of immune defence, inflammatory responses, and leucocyte migration were associated with eight DEGs (*BCL6*, *CASP4*, *CCL24*, *CCL5*, *CD247*, *IFI16*, *LBP*, *NOD1*).

The sixteen downregulated DEGs were significantly enriched with 263 GO functions, with the top 20 shown in Supplementary file Table S7B, in which 10 functions are related to the regulation of various immune processes. The regulation of chemokine production was on top (ES = 11) with three associated DEGs (*AGER*, *MCOLN2*, *ZFPM1*). Three DEGs are involved in positive and/or negative regulation of the Wnt signalling pathway (*LGR5*, *NFATC4*, *NKD1*). Four downregulated DEGs (*AGER*, *IGF2BP3*, *LGALS2*, *NFATC4*) were also associated with the regulation of biological quality, which contained the sub-functions of regulation of synaptic plasticity, RNA stability, and the homeostatic process. The regulation of RNA stability was associated with *IGF2BP3*, which was downregulated by over twofold in the MP4–7 cows.

The summarisation of the GO function with the GO browser for both up- and downregulated DEGs produced five significant GO functional categories (Table 6). They were associated with immunity (immune system process, interspecies interaction between species and response to stimulus) and the maintenance of homeostasis (biological and cellular process). Most of these functions were associated with the upregulated DEGs, indicating that they were expressed at a higher level in the older cows.

The DEGs generated in the MP4–7 vs. MP2–3 comparison were associated with 16 significant KEGG pathways with diverse functions, each containing only 2–5 mostly upregulated DEGs (Table 7). Six pathways were associated with immune/inflammatory processes (viral protein interaction with cytokine and cytokine receptor, NOD-like receptor signalling pathway, rheumatoid arthritis, influenza A, chemokine signalling pathway, and antigen processing and presentation). Two pathways (viral protein interaction with cytokine and cytokine receptor (containing *CCL24*, *CCL5*, *CCL8*) and oestrogen signalling (containing *FKBP5*, *HSPA1A*, *HSPA6*)) were also identified from the other two comparisons of MP vs. PP cows. The pathway of protein processing in the endoplasmic reticulum (*DNAJB1*, *HSPA1A*, *HSPA6*, *HSPH1*) and the NOD-like receptor signalling pathway (*CASP4*, *CCL5*, *IFI16*, *NOD1*) each contained four upregulated DEGs.

## 3. Discussion

### 3.1. Cow Longevity and Performance

Dairy cows can live over 20 years, but in practice most are culled before the end of their natural lifespan. The decision to cull cows is often involuntary due to infertility or diseases such as mastitis and lameness, but it also has an economic element, as individuals must remain profitable. The average productive lifespan in countries with high-producing dairy cows is approximately 3 to 4 years after first calving [1,8]. Longevity has declined over the past 50 years, a trend which is negatively correlated with the rise in milk yields achieved over the same time period [1]. Although selection indices have been altered more recently to increase the emphasis on health and survival traits [35], poor longevity still remains a cause for concern. The exact ages of the cows used in this study were not recorded, but the expectation would be for the PP cows to be between 2 and 2.5 years old, MP2–3 between 3.5 and 6 years, and MP4–7 between 6 and 10 years. Only 8 out of 168 cows available for recruitment (4.8%) were in lactations 6 and 7, and none had reached their eighth lactation, whereas over half of the cows were in lactations 2–3. Most cows entering these five herds were therefore culled before they achieved their peak milk production potential, suggesting that their optimum productive life was not achieved.

Comparing the three lactation groups, the average body weights for PP, MP2–3, and MP 4–7 cows were 534, 634, and 686 kg, respectively. The PP and MP2–3 cows had therefore reached around 78% and 92% of their mature weight, as there was little increase in BW beyond 5 years of age [36]. As the PP cows were not yet physically mature, they required energy and nutrients for their own continued growth during their first lactation [36,37]. Average milk yields at around 14 DIM also increased with age (PP, 23 kg/d; MP2–3, 35 kg/d; MP4–7, 37 kg/d), indicating that the greatest rise in yields occurred between the first and second/third lactations. These differences were reflected in the concentrations of metabolites measured. There were decreases in the concentrations of circulating glucose and IGF-1 between the PP and MP cows, while the differences between the two MP age groups were relatively small. This suggests that the milk yield capacity was not fully developed in PP cows and there was less uncoupling of the somatotrophic axis, together with reduced nutrient partitioning towards milk production [37,38]. The older cows had higher concentrations of NEFAs and cholesterol, suggesting that they experienced more severe mobilisation of body lipids after calving. Metabolic disorders are one of the major risk factors causing the culling-related symptoms and diseases in dairy cows [11,14]. In addition, metabolism and cellular senescence interact with each other: metabolic disorders promote senescence, which in turn causes metabolic diseases [39]. The older cows in this study did indeed experience more health issues in early lactation, including both infectious (metritis, mastitis) and metabolic (milk fever, displaced abomasum) diseases; the overall incidence rose from 9.7% in PP cows to 32.5% in MP4–7 cows.

These phenotypic differences agreed with the changes in hepatic transcriptomic profiles in terms of the numbers of DEGs. In the comparisons between the older and younger cows (MP4–7 vs. PP, MP2–3 vs. PP and MP4–7 vs. MP2–3), 719, 568, and 82 DEGs were identified, respectively. Most of the differences in gene expression profiles were, therefore, found in the comparisons between the MP and PP cows. The ongoing competition between milk production, body maintenance, and other essential functions such as immune protection is altered as cows age, and associated with a redistribution of available energy and nutrient supplies between their cells, tissues, and organs. A better understanding of the biology of ageing should help to elucidate the mechanisms underlining the relatively short productive life of dairy cows. The liver is a major organ for filtration, digestion, metabolism, detoxification, protein synthesis, and the storage of vitamins and minerals, and plays important roles in body defence [40,41]. To our knowledge, this is the first study to compare the hepatic global gene expression profiles in early lactation associated with increasing lactation number.

### 3.2. Mechanisms Underlying the Ageing Process

The ageing process is characterised by many alterations at molecular, cellular, and tissue levels, associated with a progressive loss of physiological integrity and increasing susceptibility to disease [32,42,43]. Gene transcription studies in both human populations [44] and model organisms [45,46,47] have identified signatures of the ageing transcriptome which occur repeatedly across different tissue types [30,31]. Many aspects of immune function alter during ageing, eventually leading to immunosenescence [31]. The baseline level of systemic inflammation increases (termed “inflammaging”), driven by a number of mechanisms including the accumulation of misfolded proteins, impaired clearance of senescent cells, and obesity [48,49]. Previously identified genes associated with cellular ageing have been classified into six main categories. In brief, these are the (i) downregulation of genes encoding mitochondrial proteins; (ii) downregulation of the protein synthesis machinery (including ribosome biogenesis); (iii) dysregulation of immune system genes; (iv) reduction in growth factor signalling; (v) constitutive responses to stress and accumulated DNA damage; and (vi) dysregulation of processes regulating gene expression and mRNA processing (transcription and translation) [30,31]. However, these findings cannot simply be extrapolated to dairy cows for two main reasons. Firstly, genetic selection for high milk production has placed a great metabolic demand on the body, and secondly their lifespan is also associated with their capacity to generate an economic profit.

Bovine leucocytes obtained in early lactation found evidence that many of the same genes and pathways discovered in model organisms were associated with an increased lactation number in dairy cows [34]. In humans, the deterioration of liver function is also an important symptom of the ageing process [50]. The present study provides evidence that age-related changes are also occurring in the bovine liver and are associated with the high metabolic demands of lactation. About 42–52% of DEGs derived from all three comparisons of the older cows with the younger ones were involved in the GO function of biological regulation. The predominantly downregulated DEGs play roles in regulating various important biological processes, as discussed below.

#### 3.2.1. Digestion and Absorption Processes

Many pathways related to digestion and absorption were identified in the MP cows, including those of proteins, lipids, short chain fatty acids, and vitamins, with genes in these pathways being mainly downregulated in the older animals. The liver processes nutrients absorbed from the small intestine and transported in the hepatic portal vein, while bile secreted into the duodenum plays an important role in emulsifying lipids and digesting some vitamins. The GO function of digestion topped the list of the multicellular organismal process in both MP age groups, and contained four downregulated DEGs (*ASAH2*, *LYZ2*, *LYZ3*, *TMPRSS15*). ASAH2 (N-acylsphingosine amidohydrolase 2) is essential for the intestinal degradation of sphingolipids [51], and TMPRSS15 (transmembrane serine protease 15) is responsible for initiating the activation of pancreatic proteolytic proenzymes (trypsin, chymotrypsin, and carboxypeptidase A) [52]. Lysozyme (LYZ) facilitates the digestion of bacteria and is recognised as an innate immune defence factor, providing protection against bacteria, viruses, and fungi [53]. *PRSS1*, encoding serine protease 1 and the one upregulated gene in this category, is involved in the production of cationic trypsinogen, another digestive enzyme.

#### 3.2.2. Protein Synthesis

The maintenance of the proteome is essential to enable cells to respond appropriately to their environment. This requires the correct synthesis and assembly of proteins in the endoplasmic reticulum, and is controlled by molecular chaperones and clearance mechanisms that help to prevent protein misfolding and the associated accumulation of toxic aggregates. The efficiency of this process declines with age, and has previously been associated with both metabolic and immunological diseases [54,55]. Heat-shock proteins (HSPs), which function as molecular chaperones, are upregulated under conditions of stress in which the concentrations of aggregation-prone folding intermediates increase [56]. The present study showed that *DNAJB1* (HSP40 member B1), *HSPA1A*, *HSPA2*, *HSPA6*, and *HSPH1* were all expressed at higher levels in the MP4–7 cows compared with the PP cows, while *DNAJC18* (HSP40 member C18) was expressed at a lower level. Previous studies reported that the upregulation of *HSPA1A*, *HSPA2*, and *HSPA6* was associated with mitochondrial damage [57,58]. *FBXW5* was also upregulated in the MP4–7 cows. This encodes F-box/WD repeat-containing protein 5, a member of the FBXW subclass of F-box proteins, which functions in phosphorylation-dependent ubiquitination and may play a role in autophagy. The differential expression of this gene was previously demonstrated in the hepatic transcriptome of dairy cows, according to the type of forage fed [59]. An alteration in protein maturation was identified in the GO enrichment analysis in both MP cow groups compared with the PP group. This involved ten downregulated (*ADAM19*, *ADAMTS2*, *CASP1*, *GP1BA*, *NLRP6*, *P2RX7*, *PLAT*, *PYCARD*, *SERPINH1*, *TMPRSS15*) and two upregulated (ECE2, *IL1R2*) DEGs. Maturation is vital for a protein to attain its full functional capacity. Its alteration may lead to the loss or dysfunction of proteostasis, which is associated with the ageing process [60].

#### 3.2.3. Metabolism

The liver is a major centre for nutrient metabolism, and the deterioration of liver metabolic capability is an important symptom of the ageing process [50]. The GO biological function of the metabolic process derived from the MP4–7 vs. PP comparison was associated with over 200 DEGs playing roles in various metabolic processes involving collagen, primary small molecules, organic substances, hormones, nitrogen compounds, and NADH (the reduced form of nicotinamide adenine dinucleotide, important for cellular energy production). Many GO functions associated with both the synthesis and catabolism of various molecules were also enriched with upregulated DEGs (Figure 3A,C). The primary metabolic process was altered by 121 downregulated and 61 upregulated DEGs (Supplementary file Table S8A), playing roles in the processing of lipids, carbohydrates, proteins, and amino acids). KEGG pathway analysis similarly identified the bta01100:Metabolic pathway as the most important in the comparisons of both MP4–7 (ES = 13 with 82 associated DEGs, Supplementary file Table S8B) and MP2–3 (ES = 10 with 66 associated DEGs, Supplementary file Table S8C) with PP cows.

A further analysis of these pathways showed that the DEGs identified under lipid metabolism (ES = 7, associated with 28 downregulated and 12 upregulated DEGs, Supplementary file Table S8D) were primarily involved in the metabolism of glycerophospholipids (*ENPP6*, *GAL3ST1*, *GLYCTK*, *GPAT3*, *LOC615045*, *PLA2G1B*, *PLA2G4F*, *PLB1*, *PLD4*), all of which were downregulated in the older cows. Glycerophospholipids form the main lipid component of cell membranes, contributing to their stability, fluidity, and permeability, and to the proper functioning of membrane proteins, receptors, and ion channels [61]. Other DEGs encoding lipases and phospholipases are involved in fat digestion (*CEL*, *LPIN1*, *LOC615045*, *MGLL*, *PLA2G4F*, *PNLIP*, *PNLIPRP2*), of which three (*CEL*, *PNLIP*, *PNLIPRP2*) were more than 9-fold reduced in the MP4–7 cows. *SLC27A4* (also downregulated) encodes a fatty acid transport protein, which translocates long-chain fatty acids cross the plasma membrane. Previous studies have similarly illustrated that dysregulated lipid metabolism is associated with ageing [62,63], and problems with lipid metabolism are well known to compromise the health of periparturient cows [64].

Of the genes identified as having a role in carbohydrate metabolism (ES = 8, associated with 16 downregulated and 4 upregulated DEGs, Supplementary file Table S8E), *PC* (encoding pyruvate carboxylase) and *FBP2* (encoding fructose-bisphosphatase 2) were both upregulated in the MP4–7 vs. the PP cows. When nutrient supply is limiting, as happens when there is a large nutrient drain into milk production, hepatic pyruvate is primarily generated from lactate and alanine as opposed to glucose, and the pyruvate is then converted to oxaloacetate by pyruvate carboxylase, the first step in gluconeogenesis. This switch occurs primarily in response to an increase in free fatty acid oxidation [65]. *FBP2* encodes fructose-bisphosphatase 2, which catalyses the hydrolysis of fructose 1,6-bisphosphate to fructose 6-phosphate. This acts at a key point in controlling the flux between glycolysis and gluconeogenesis, and its expression was 5.1-fold higher in the MP4–7 than the PP cows, which would favour gluconeogenesis. Conversely, two genes involved in glycogen synthesis (*PPP1R3*, *BPPP1R3C*) were downregulated in the older cows. Two other interesting DEGs, which were both downregulated in the MP4–7 vs. PP cows, were *PDX1* and *C1QTNF3*. *PDX1* encodes the transcriptional activator pancreatic and duodenal homeobox 1, the expression of which was 7.6-fold lower in the older cows. The encoded nuclear protein regulates a number of key genes involved in glucose homeostasis including insulin, somatostatin, glucokinase, and glucose transporter type 2, and it plays a major role in the glucose-dependent regulation of insulin gene expression by the pancreas [66]. *C1QTNF3* encodes C1q and TNF related 3, a secreted protein which has a glucose-lowering effect. It was shown to act independently of insulin to regulate gluconeogenesis in hepatocytes by suppressing the expression of two key gluconeogenic enzymes, glucose-6-phosphatase and PEPCK, by activating the PKB/Akt signalling pathway [67]. These changes suggest a differential hepatic control of energy metabolism as the cows’ milk production capacity increases with age. Circulating glucose concentrations were significantly lower in the older cows, and it appears from these results that this was driving the liver to increase gluconeogenesis, although it should be noted that neither *G6PC* nor *PEPCK* expression were themselves altered in the different lactation groups.

Protein metabolism (ES = 3, associated with 61 downregulated and 31 upregulated DEGs, Supplementary file Table S8F) included some DEGs that encode enzymes having a direct role in protein breakdown, and which are also produced in the pancreas, e.g., *CELA2A*, *CPA1*, and *PRSS1*. A number of other DEGs are involved in the NOD-like receptor signalling pathway, which is discussed below in the section on immune function. The catabolism of most amino acids begins in the liver, and pathways of amino acid processing (ES = 9, associated with 6 downregulated and 10 upregulated DEGs, Supplementary file Table S8G) were also identified. Of these, *ARG1* was upregulated in the MP4–7 cows. This encodes arginase type 1, a cytosolic enzyme expressed predominantly in the liver that catalyses the hydrolysis of arginine to ornithine and urea as the final component of the urea cycle. *ANDHD1*, *HAL*, and *HDC* (all upregulated in the MP4–7 cows) encode enzymes that are involved in the catabolism of histidine and its conversion to histamine, which is released from mast cells. Hepatic mast cells are mainly associated with the connective tissue surrounding the blood vessels and bile ducts; their number increases during hepatic injury and fibrosis, suggesting that they play a role in liver disease [68].

The expression of *GCLC*, encoding the first rate-limiting enzyme of glutathione synthesis, was 1.4-fold lower in the older animals, whereas *GPX3*, encoding glutathione peroxidase 3, was upregulated by 2.9-fold. Several genes encoding glutathione-S-transferases (GST) were also differentially expressed, with *GSTM1_2*, *GSTM2* and *GSTM3* all being upregulated in the older cows, whereas *GSTP1_2* was downregulated. *LOC615514*, encoding glutathione S-transferase mu 1-like, was also upregulated in the MP cows and was one of the DEGs which differed between all three age groups (Supplementary file Table S1). Glutathione is an important antioxidant that exists in cells mainly in a reduced state (GSH). Reactive oxygen species (ROS) are generated in mitochondria during lipid metabolism, leading to lipid peroxidation, membrane damage, and ultimately cell death. Glutathione peroxidase reduces hydrogen peroxide to water, using GSH as a cofactor, thus helping to maintain membrane integrity and minimise ROS-incurred damage [69]. GSTs are a group of phase II detoxification enzymes that metabolise a variety of substrates to form highly hydrophilic and less chemically active compounds, which can then be excreted through bile. Their expression can be upregulated by ROS and their abundance is considered as an important factor in determining the sensitivity of cells to a range of toxins [70]. In human populations, polymorphisms in GSTs have been associated with a number of liver diseases [70]. Changes in the expression of these various glutathione-related enzymes suggest that these important protective processes are altered in the livers of older cows.

#### 3.2.4. Immune Function

The liver plays important roles in body immunity by providing a surveillance system for pathogens, synthesising and secreting many inflammatory mediators. These include complement, coagulation proteins, acute phase proteins, and inflammatory cytokines, which are all important components of non-specific innate immunity and play roles in restoring homeostasis and host defence mechanisms against invading microorganisms and inflammation [24,25,40,41]. Kupffer cells (KC), a population of resident macrophages, comprise nearly one-third of non-parenchymal cells in the liver [71]. A wide variety of lymphocyte populations are also transiently or permanently located in the liver, including natural killer (NK) cells, and various types of T-cells and B-cells [72,73,74]. Hepatocytes and liver sinusoidal endothelial cells also exhibit immunological functions [75,76].

As animals age, they experience a progressive loss of immune function which increases their vulnerability to infection [42]. Notable age-related changes within the general immune cell population include reduced cytokine signalling, diminished production of nitric oxide and peroxide, decreased phagocytic ability, and reduced ability of dendritic cells to migrate and process antigens [77,78]. Mounting an immune response requires an adequate supply of glucose, various fatty acids, and cholesterol or oxysterols [16,79]. For cows in early lactation, there is competition for the allocation of energy and nutrients between milk production and immunity; this is exacerbated in older cows, as their increased milk production capacity causes a greater drain on glucose availability [14,16]. In our previous study of circulating leucocyte populations, we reported that those from PP cows showed an upregulation of genes associated with T-cell development and function, while genes upregulated in MP > 3 cows included those encoding proteins involved in combatting disease pathogens through the activation of the innate immune system [34].

The present results from the liver also show differential immune responses associated with lactation number. Both GO and pathway analyses indicated that genes involved in the response to bacteria and regulation of cytokine production were expressed at a higher level in the PP cows in comparison to the two MP groups. These included, for example, *IFI44*, *JCHAIN*, *LEAP2*, *LCN2*, *LYPD8*, and *REG4*. Most genes associated with bta04621, the NOD-like receptor (NLR) signalling pathway, were expressed at higher levels in PP cows, including *CASP1*, *NLRP6*, *P2RX7*, *PYCARD*, *PRKCD*, and *RIPK3*, whereas *NOD1* was upregulated in the older cows. The intracellular NLR family plays a pivotal role in the recognition of intracellular ligands including bacterial peptidoglycan fragments, DNA, and ssRNA viruses. This can drive the activation of NFκB and MAPK, leading to the production of cytokines, chemokines, and antimicrobial peptides (AMPs). A different set of NLRs induces caspase-1 activation through the assembly of multiprotein complexes called inflammasomes, whose formation can be triggered by infections, tissue damage, or metabolic imbalances. The activated caspase-1 regulates the maturation of the pro-inflammatory cytokines IL-1B, IL-18, and drives cell death via pyroptosis. This signalling system is known to be associated with hepatic inflammation [80].

Two other genes encoding lysozyme were highly downregulated in the comparison between MP4–7 and PP cows (*LYZ2* by 2.9-fold and *LYZ3* by 6.4-fold). This enzyme is produced by Kupffer cells within the bovine liver [81]. It breaks down bacterial cell walls and is recognised as an innate immune defence factor, providing protection against bacteria, viruses, and fungi [53]. The pathways of arachidonic acid and linoleic acid metabolism were also differentially regulated in the MP4–7 cows. Of the DEGs identified, *PLA2G1B PLA2G4F*, and *PLB1* were all downregulated. These encode phospholipases that could release arachidonic acid from the plasma membrane. *LTC4* (downregulated) and *GGT1* (upregulated) encode enzymes involved in the conversion of leukotriene C4 to leukotrienes D4 and F4. These leukotrienes may potentially be involved in inflammatory responses in mast cells [82].

On the other hand, some genes with pro-immune and inflammatory properties were upregulated in the older cows compared with the younger ones. For example, in the MP4–7 vs. MP2–3 comparison, the GO function of the immune system process was on the top and was associated with eight upregulated DEGs, including *NOD1*, *LBP* (encoding lipopolysaccharide binding protein), and *PTX3* (Table 6). *PTX3* encodes pentraxin 3, a well-recognised biomarker for inflammatory conditions including liver disease, which is involved in complement activation, angiogenesis, and tissue remodelling [83]. Two other important antimicrobial peptide genes (*HAMP*, *LTF)* were also upregulated in the MP4–7 cows compared with PP cows. *ARG1* and *ARG2*, encoding arginase 1 and 2, were also upregulated. While arginase 1 is highly expressed in the liver and is involved in the urea cycle, *ARG1* expression is also a signature gene used to define alternatively activated M2 macrophages, as opposed to classically activated M1 macrophages, which express iNOS [84]. Another interesting difference is that M2-polarised cells exhibit enhanced oxidative phosphorylation, and preferentially use glutamine and fatty acids as an energy source in comparison to M1 macrophages, which rely on glucose [85]. M2 macrophages produce immunosuppressive factors which inhibit the development and proliferation of many types of immune cells, and reduce responsiveness to inflammatory mediators, phenotypes associated with immunosenescence [78].

The downregulation of some aspects of hepatic immune function in cows from their second lactation onwards suggests that their increase in milk production in early lactation compromises immune function due to the competition for nutrients. This, in turn, would predispose these older animals to mastitis and uterine disease [86,87,88,89], both major risk factors leading to culling [9,90]. On the other hand, antimicrobial peptides were expressed at a higher level in the older cows, possibly in response to such infections.

#### 3.2.5. Growth Factor Signalling

After calving, most dairy cows experience a period of NEB that is associated with insulin resistance, reduced hepatic growth hormone (GH) receptor expression, decreased hepatic synthesis of IGF-1, and altered expression of most of the IGF binding proteins [27,91]. These changes act to prioritise the available glucose supply to the mammary gland for milk synthesis at the expense of growth and tissue repair [16,92]. In accordance with this, hepatic *IGF1* and *IGFALS* mRNA expressions were both lower in MP4–7 vs. PP cows, while *IGFBP2* mRNA was increased. These changes would reduce the bioavailability and increase the clearance of IGF-1 from the circulation [27]. *VEGFC* expression was also lower in both MP groups. This is a member of the platelet-derived growth factor/vascular endothelial growth factor (PDGF/VEGF) family. The encoded protein promotes angiogenesis and endothelial cell growth and can also affect the permeability of blood vessels. There is some evidence that VEGFC could help to regulate the vascular supply to the hepatic biliary tree, which is essential in supporting the secretory and absorptive functions of the biliary epithelium [93].

#### 3.2.6. Responses to DNA Damage, Gene Expression, and mRNA Processing (Transcription and Translation)

Genome instability is one of the hallmarks of ageing in the liver [33]. The MAPK dual specific phosphatase (DUSP) modulates the nucleotide excision repair pathway and cell cycle regulatory proteins to maintain genomic stability and cell proliferation [94]. It is also involved in regulating T-cell senescence/exhaustion and chronic immune-related disease [95]. In the present study, *DUSP3* was in the top 10 list of most significantly upregulated genes in both age groups of MP cows compared with PP cows. Its activation suggests that the older cows experienced stress, affecting genome homeostasis.

Cellular senescence involves cell cycle arrest in damaged or aged cells, which can also be triggered in normal cells in response to various intrinsic or extrinsic stimuli and developmental signals [96]. Cyclin-dependent kinases (CDKs) are the key enzymes regulating cell proliferation through controlling cell cycle checkpoints and transcriptional events, and their catalytic activity is regulated by their interaction with cyclins and CDK inhibitors [97]. *CDKN2A* encodes the CDK inhibitor p16^INK4a^ which plays crucial mechanistic roles in the implementation of the senescent programme, by halting the cell cycle through the p16^INK4a^/Rb pathway, and has been widely used as a genetic marker of cellular senescence in vivo [98,99]. In the present study, the expression of *CDKN2A* was upregulated in all three comparisons of the older cows with the younger ones, indicating accelerating cellular senescent processes in the older cows.

Both *IGF2BP2* and *IGF2BP3* mRNA expression was downregulated in the older cows, with *IGF2BP3* being the most significant DEG in the comparisons of MP4–7 cows with both the PP and MP2–3 groups. These genes both encode members of a family of mRNA binding proteins that are involved in a spectrum of biological processes which play major roles in maintaining RNA stability and post-transcriptional regulation, and are important in coordinating nutrient stimulation with RNA life cycle control [100]. IGF2BP2 is associated with impaired insulin secretion and human type 2 diabetes. Studies in mice have shown that it regulates fatty acid oxidation through the post-transcriptional regulation of gene expression across multiple tissues, including the liver; IGF2BP2-deficient mice have improved glucose tolerance and insulin sensitivity, although their pancreatic islets secrete less insulin than in control animals [100]. IGF2BP3 plays a role in controlling the trans differentiation of hepatic stellate cells into myofibroblasts [101]. DNA replication was inhibited in cells in which the *IGF2BP3* gene was knocked down [102]. The present study suggests that IGF2BP3 is a potential biomarker for hepatic ageing in dairy cows.

#### 3.2.7. Hepatic Morphology

Regressive changes in tissue and cell morphology are one hallmark of the ageing process, with cells potentially becoming enlarged, flat, multivacuolated, and/or multinucleated [103,104]. Many morphological changes in hepatic cells were reported previously in both human and model animals. These included increased polyploidy, the accumulation of lipofuscin in the cytoplasm, a declining surface area of endoplasmic reticulum, and a reduced number of mitochondria, all ultimately having a negative effect on hepatocyte function [105,106]. The present study did not directly examine the morphology of the liver samples, but the analysis showed that the expressions of many genes playing roles in structure and morphology were altered. The GO enrichment analysis illustrated that 11 biological functions related to the extracellular matrix were in the top 20 list in the MP4–7 cows compared with the PP cows (Figure 3B), and involved 61 downregulated DEGs (Supplementary file Table S3A). The GO browser summarised the function of the multicellular organismal process, and also included the altered sub-function of morphogenesis of a branching structure by the downregulated DEGs in both MP groups compared with the PP cows.

Many key genes involved in collagen synthesis were expressed at a lower level in MP4–7 compared with PP cows (*ADAMTS2*, *COL1A1*, *COL1A2*, *COL3A1*, *COL4A5*, *COL5A1*, *COL5A2*, *COL12A1*, *COL15A1*, *LOX*, *LOXL2*, *MMP2*, *PLOD2*, *SERPINH1*), and most of these were also significant in the MP2–3 vs. PP comparison. This implies that the PP cows were developing hepatic fibrosis at the start of their first lactation, confirming our previous report based on a smaller number of animals [107]. Studies in humans have indicated that this process follows the activation of hepatic stellate cells (HSCs), which are mainly responsible for remodelling the extracellular matrix [108,109], and this is usually preceded by inflammation. The HSCs then differentiate into myofibroblasts (MFB), which synthesise both fibrils, forming collagen, and a variety of other extra-cellular matrix proteins. TGFβ is released from the latent TGF-β binding protein complex during fibrosis when linkages form between the extracellular matrix and cytoskeleton, and this is considered to be a major factor in hepatic stellate cell activation, thus accelerating liver fibrosis [110]. In this study, both *TGFA* and *TGFB3* were expressed at higher levels in the PP cows, although the expression of *TGFB2* was greater in the MP4–7 group. The liver has to undergo significant adaptation at the start of lactation when both its metabolic activity and blood flow have been estimated to double [111,112,113]. These changes might trigger hepatic collagen deposition at the start of the first lactation, but it remains to be determined whether such a change is temporary or permanent. As well as affecting tissue structure, collagen is also a long-established immune enhancer involved in many immune/inflammatory processes [114].

Vitamin D is a pleiotropic hormone with regulatory functions in calcium homeostasis, inflammation, and metabolism. Rodent models have shown that it can reduce hepatic inflammation, oxidative stress, and insulin resistance, while in humans hepatic *VDR* expression is inversely correlated with the severity of steatosis and inflammation. Furthermore, vitamin D exerts anti-fibrotic activity by inhibiting the proliferation of hepatic stellate cells, and it can also modulate intra-hepatic lipid accumulation [115]. In this study, *VDR* expression was 2-fold higher in the PP cows, which may have contributed to their apparent higher rate of collagen deposition.

#### 3.2.8. Nutrient Transport

In the present study, the DEGs derived from all three comparisons of the higher lactation number cows with the lower ones were associated with the GO term of transport (97 in MP4–7 vs. PP, 81 in MP2–3 vs. PP and 15 in MP4–7 vs. MP2–3 comparisons, (Supplementary files Tables S3I, S5J and S7C)). This term refers to the directed movement of substances such as macromolecules, small molecules, and ions or cellular components (such as complexes and organelles) into, out of, or within a cell or between cells. Fiore et al. [116] previously reported an age-related change in the expression of the facilitated glucose transporter *SLC2A4*, which was higher in the muscle of older dairy cows. The present study identified 22 genes encoding solute carrier transporters (SLC), the majority of which were downregulated in the livers of MP4–7 cows, including those for glucose (*SLC5A10*), glucose-6-phosphate (*SLC37A2*), amino acids (*SLC1A4*, *SLC3A1*), peptides *(SLC15A1*), fatty acids (*SLC27A4*), nucleosides (*SLC28A1*, *SLC29A2*), prostaglandins (*SLCO2A1*), folate (*SLC46A1*), sulphates (*SLC13A1*), Na+/H+ (*SLC9A1*), and organic anions and cations including toxins (*SLC22A2*, *SLC22A7*, *SLCO4A1*). A smaller number of transporters were upregulated, for glutamate (*SLC1A2*), GABA (*SLC6A11)*, metabolites such as citrate and urate (*SLC13A2*, *SLC13A5*, *SLC17A1*), and organic anions (*SLC29A2*). This concurs with previous studies, showing that the capability of sensing nutrients and transporting them to their appropriate subcellular location decreased in senescent cells [117,118,119]. The expression of three genes encoding vacuolar ATPases (*ATP6V0A4*, *ATP6V1B1*, *ATP6V1C2*) was higher in MP4–7 than PP cows. These encode enzymes that mediate the acidification of intracellular compartments. Four genes encoding potassium channels in the plasma membrane were also differentially expressed: *KCNH7* and *KCNJ15* were upregulated in the older cows, whereas *KCNJ4* and *KCNT2* were downregulated. Both intra- and extra-cellular ion transport also play a major role in establishing senescence [120,121].

#### 3.2.9. Oestrogen Signalling

The oestrogen signalling pathway was altered in all three comparisons of the older cows with the younger ones. Classical oestrogen signalling occurs via nuclear receptors. In the absence of a ligand, these require an association with chaperones to maintain proper folding of the ligand-binding domain, and the heat shock protein 70 (HSP70) chaperone machinery is essential for a proper response to steroids [122]. *HSPA1A*, *HSPA2*, and *HSPA6*, which all encode for HSP70 members, were all upregulated in the older cows. The other genes identified as contributing to the oestrogen signalling pathway (*CREB3L1*, *CREB5*, *GABBR2*, *GNAO1*, *KRT20*, *MMP2*, *PRKCD*, *SRC*, *TGFA*) were all downregulated in the older animals. Apart from *TGFA*, these participate in membrane-initiated signalling pathways, and could therefore involve membrane rather than nuclear located oestrogen receptors. Oestradiol can regulate the genes involved in hepatic lipid and glucose metabolism, and is considered protective against liver disease in female mammals [29,123]. However, *GABBR2* encodes a subunit of the GABA-B receptor subfamily and GNAO1 acts downstream of this. There is evidence from rodent models that hepatic lipid accumulation favours GABA production, and that its release is promoted by hepatocyte depolarisation, which is stimulated by K+ efflux [124]. As just mentioned, the GABA transporter *SLC6A11* and four genes encoding potassium channel family members were all differentially expressed between MMP4–7 and PP cows. In diabetic humans, GABA treatment can reduce circulating glucose and improve insulin resistance [125].

## 4. Materials and Methods

### 4.1. Animals and Blood Sampling

Holstein Friesian cows were recruited from five experimental dairy farms located in different EU countries, including Agri-Food and Biosciences Institute Hillsborough (*n* = 58, Northern Ireland, UK), Aarhus University (*n* = 34, Denmark), University College Dublin (*n* = 38, Ireland), Leibniz Institute for Farm Animal Biology (*n* = 13, Germany), and Walloon Agricultural Centre (*n* = 25, Belgium). All procedures, including the blood sample collection and invasive collection of liver biopsy, had local ethical approval (See Section “Institutional Review Board Statement” for more details) and complied with the relevant national and EU legislation under European Union Directive 2010/63/EU.

All cows recruited in this study were regularly checked by the veterinarians and staff on each farm, and the health status for each cow was recorded as described previously [126]. Calving ease was scored on a scale of 1 (easy calving, no help) to 5 (caesarean section). The cows in two of the herds (Agri-Food and Biosciences Institute, UK and Aarhus University, Denmark) received three contrasting diets, which were balanced for lactation numbers between the dietary groups, whilst the cows in the remaining three herds were offered diets which reflected the local management practice. Further details of the management and lactation diets of each herd were described previously [126,127]. At 14 ± 2 DIM, blood samples were collected after morning milking to obtain plasma (in Na heparin tubes) and serum (plain tubes) for an analysis of circulating metabolites and IGF-1. Plasma and serum were separated by centrifugation (1600× *g* at 4 °C for 15 min) and stored at −20 °C for subsequent analysis.

### 4.2. Animal Grouping Based on the Lactation Number

A total of 168 dairy cows were grouped based on their lactation number: (1) PP (primiparous cows, lactation 1, *n* = 41), (2) MP2–3 (multiparous cows, lactations 2–3, *n* = 87), and (3) MP4–7 (multiparous cows, lactations 4–7, *n* = 40).

### 4.3. Analysis of Circulating Metabolites and IGF-1

Concentrations of glucose, urea, BHB, NEFAs, and cholesterol were measured using the methods described previously [20,28]. Intra- and inter-assay coefficients of variation were in all cases below 3% and 4%, respectively, for the samples from the three lactation groups. The concentrations of IGF-1 were determined in serum by radioimmunoassay following acid-ethanol extraction [128]. Intra-assay coefficients of variation (CV) were 12.4, 7.5, and 9.9% for low, medium, and high concentration control samples, respectively.

### 4.4. Cow Phenotype Data Collection

Body weights were recorded twice weekly using weigh scales. Body condition scores were estimated according to a common protocol at 14 ± 2 DIM (mean ± SD), using a five-point scale with quarters [129]. All cows were milked twice daily, and their daily yields were recorded. The concentrations of protein and fat in milk were quantified with mid-infrared analysis. ECM (kg/day) was calculated following the methods reported previously [127]. On four of the five farms, daily DMI were recorded using electronic feeding systems. EBAL was calculated as described previously [126].

### 4.5. Liver Biopsy Sampling and RNA Extraction

Liver biopsies were taken from all selected cows at 14 ± 2 DIM, using a standard operating procedure described previously [130], and for each cow both blood and liver biopsy samples were collected at the same hour of the day. The samples were snap-frozen in liquid nitrogen and stored at −80 °C until RNA extraction. Total hepatic RNA was extracted following the method described previously [130]. Briefly, 20 mg of liver tissue was fully homogenised using a TissueLyzer II homogeniser in 600 µL Buffer RLT with a 5 mm stainless steel bead (QIAGEN, Manchester, UK). Under a QIAcube workstation, the total RNA from the homogenate was extracted with RNeasy Mini kits following the supplier’s protocol (QIAGEN). The quality and quantity were assessed with a QIAxcel capillary electrophoresis device (QIAGEN) and NanoDrop 2000 spectrophotometer (Thermo Fisher Scientific, Waltham, MA, USA). This showed average RNA concentrations of 309 ± 113 ng/µL, a range of absorbance ratios of 1.98–2.14, and an RNA integrity score of 7.8 ± 0.7. The details are given in Supplementary file Table S9. The RNA was stored at −80 °C until subsequent RNA-sequencing.

### 4.6. RNA-Sequencing, Mapping, and Quantification

The RNA-sequencing was carried out on an Illumina NextSeq 500 platform as described previously [86]. Briefly, the sequencing libraries were prepared with 750 ng of the total RNA extracted as described above, and the Illumina TruSeq Stranded Total RNA Library Prep Ribo-Zero Gold kit (Illumina, San Diego, CA, USA) in the epMotion liquid handling workstation (Eppendorf, Hamburg, Germany). The pooled cDNA libraries were sequenced on the Illumina NextSeq 500 sequencer at 75 nucleotide length single-end reads with an average of 30 million reads per sample. The raw FASTQ files were deposited to the European Nucleotide Archive (ERP124149, ERP125646).

The FASTQ files sequenced from different lanes for each sample were merged into one file. A CLC Genomics Workbench V21 (QIAGEN Digital Insights, Redwood City, CA, USA) was used for sequencing analysis, including trimming the poor reads and quality control, and mapping the reads to a reference genome of *Bos taurus* assembly (ARS-UCD1.2, supplied by RefSeq at https://www.ncbi.nlm.nih.gov/assembly, accessed on 1 January 2021). The sequencing data were quantified as reads per genes and reads per kilobase of transcript per million mapped reads (RPKM) in the formats of gene expression (GE) files.

### 4.7. Analysis of Differential Gene Expression between the Dietary Groups

Differential gene expression was carried out using CLC Genomics Workbench V21 with the GE files. The gene expression values were normalised with trimmed mean and Z-score methods across all samples. Principal component analysis showed that there was a difference in the overall gene expression pattern between the herds (Supplementary file Figure S1A). To account for this during the analysis of differentially expressed genes (DEGs) between the lactation groups, a two-way analysis of variance (ANOVA)-like model was used with age group as the test variable and herds as the confounding control variable, which minimised the herd effect (Supplementary file Figure S1B). The Benjamini–Hochberg (BH) procedure was used to control the false discovery rates (FDR) against errors due to multiple testing, and significance was considered at *p* < 0.05. Where the expression value of the higher lactation number was greater than that of the lower lactation number (positive fold change, upregulation), the fold changes (FC) were calculated as the gene expression ratio of the higher lactation number group to the lower lactation number group (i.e., MP4–7 vs. PP, MP2–3 vs. PP, or MP4–7 vs. MP2–3). Where the expression value of the lower lactation number group was greater than that of the higher lactation number group (negative fold change, downregulation), the ratios were calculated as PP vs. MP4–7, PP vs. MP2–3, or MP2–3 vs. MP4–7) with a minus in front. The cut off criteria were set to an absolute FC ≥ 1.25 and FDR (BH) *p* < 0.05. The genes meeting these criteria in pairwise comparisons among the three groups were selected for subsequent analysis.

### 4.8. Gene Ontology Enrichment Analysis

Gene Ontology (GO) and pathway enrichment analyses (with Kyoto Encyclopedia of Genes and Genomes (KEGG)) were performed to investigate the biological functions and interactions between genes and pathways using Partek Genomics Suite V7 (Partek Incorporation, Chesterfield, MO, USA), with a genome version of ARS-UCD1.2. The DEGs were used for pathway enrichment analysis and GO enrichment analysis. The GO enrichment analysis was performed using both the total DEGs and separately for up- and downregulated DEGs, and it focused on biological processes, while only total DEGs were used for pathway enrichment analysis. Fisher’s exact test with BH adjustment for GO enrichment and q-value FDR for pathway enrichment were used, and statistical significance was considered at *p* < 0.05. The enrichment score (ES) was calculated as -ln (*p*) so that an ES >3 was considered as significant.

### 4.9. Statistical Analysis

The statistical analysis of phenotype data was carried out using IBM SPSS V29 (IBM Corp., Armonk, NY, USA) to test the differences between BW, BCS, circulating metabolites (glucose, urea, BHB, NEFAs, and cholesterol), and IGF-1 among the three lactation groups. The data were expressed as mean ± standard error of mean (SE). The homogeneity of variance for each variable was assessed with Levene’s test before ANOVA. Logarithmic transformation was applied if the variance was not homogenous. An ANOVA with a linear mixed-effects model was used with the age group as a fixed effect and herds as a random effect. Significance was considered at *p* < 0.05. Where ANOVA showed significance, multiple comparisons with the Tukey HSD method were carried out to identify the sources of differences.

## 5. Conclusions

To the best of our knowledge, this study has shown for the first time that hepatic global gene expression profiles in early lactation cows are related to increasing lactation number (parity). This has revealed many changes in the livers of the older cows associated with a variety of biological processes and pathways, although it was not possible to separate the effects of chronological age from those of milk yield, and the cumulative stresses imposed by successive lactations. Diet is also likely to be influential, although this aspect was not examined in the present study. Most of these differences occurred between the first and second lactations, as growth slows and milk production capacity increases. This requires a greater supply of glucose, leading to a drive towards increased hepatic gluconeogenesis even though blood glucose levels were reduced. The analysis provided evidence that the livers of older cows had reduced immune capacity, dysregulated protein and glycerophospholipid metabolism, and impaired RNA stability and nutrient transport, all of which would impair functionality. Genes associated with cell cycle arrest and the production of antimicrobial peptides were upregulated. More surprisingly, evidence of hepatic inflammation leading to collagen deposition was present in the primiparous cows as they started their first lactation. The results of the study suggest that the high milk production capacity of Holstein cows has a cost in terms of liver function, which accelerates the ageing process. Disorders of liver function contribute at least in part to the short lifespans typical of modern dairy cows. Overcoming this problem requires a multifaceted approach. A better understanding of the biological processes controlling milk production should in future inform the development of both genetic selection criteria and improved nutritional management, which together should aim to reduce the metabolic stresses of early lactation. At the same time, a modelling approach is required to determine the optimum lifespan for cows which maximises economic profitability while minimising the harmful environmental impact of dairy production systems.

## Figures and Tables

**Table 1:** Blood metabolites, feed intake, bodyweight, body condition score, and milk parameters in three lactation groups on day 14 after calving ^1^.

Parameters ^2^	PP Cows	MP2–3 Cows	MP4–7 Cows
N	41	87	40
Glucose (mmol/L)	3.81 ± 0.06 ^a^	3.44 ± 0.05 ^b^	3.28 ± 0.07 ^c^
Urea (mmol/L)	3.04 ± 0.14	3.10 ± 0.15	2.71 ± 0.17
BHB mmol/L)	0.59 ± 0.07	0.70 ± 0.06	0.82 ± 0.11
NEFA (mmol/L)	623.9 ± 74.4 ^b^	697.7 ± 48.7 ^b^	881.2 ± 73.1 ^a^
IGF1 (ng/mL)	162.5 ± 13.0 ^a^	92.3 ± 5.7 ^b^	61.4 ± 7.0 ^c^
Cholesterol (mmol/L)	2.65 ± 0.08 ^b^	3.04 ± 0.07 ^a^	3.02 ± 0.11 ^a^
Total DMI (kg/d)	14.90 ± 0.39 ^b^	19.20 ± 0.43 ^a^	19.80 ± 0.67 ^a^
BW (kg)	534.7 ± 6.9 ^c^	634.5 ± 7.5 ^b^	686.6 ± 11.4 ^a^
BCS	3.10 ± 0.05 ^a^	2.83 ± 0.04 ^b^	2.86 ± 0.08 ^b^
MY (kg/d) ^3^	23.2 ± 0.9 ^c^	35.2 ± 0.7 ^b^	37.4 ± 0.9 ^a^
Milk fat (%) ^3^	4.33 ± 0.13	4.37 ± 0.08	4.53 ± 0.12
Milk protein (%) ^3^	3.28 ± 0.05	3.25 ± 0.03	3.23 ± 0.05
ECM (kg/d) ^3^	24.0 ± 0.8 ^c^	36.0 ± 0.8 ^b^	39.5 ± 0.9 ^a^
EBAL (MJ/d) ^3^	−2.6 ± 0.9 ^a^	−5.5 ± 0.8 ^a^	−8.4 ± 1.0 ^b^

^1^ Values are expressed as mean ± SE. a > b > c, *p* < 0.05 to *p* < 0.0001. ^2^ BHB: beta-hydroxybutyrate, NEFA: non-esterified fatty acid, DMI: dry matter intake, BW: body weight, BCS: body condition score (measured at both 14 and 35 days in milk), ECM: energy corrected milk, EBAL: energy balance. ^3^ Values are the average of the records over 12–16 days in milk.

**Table 2:** Summary of GO enrichment main functions of DEGs in the comparison between MP4–7 (*n* = 40) and PP cows (*n* = 41) in early lactation.

Functions	Enrichment Score	DEGs in the Function
Interspecies interaction between organisms	8.6	Downregulated DEGs (27): *ADIPOQ, ANPEP, CASP1, CCDC80, CHGA, CHMP4A, CUBN, FCER2, GSDME, IFI44, IFI44L, IL22RA1, IRF4, JCHAIN, LEAP2, NLRP6, NOD2, P2RX7, PLA2G1B, PNLIPRP2, PRKCD, PYCARD, REG4, RIPK3, S100A14, SARM1, VIL1 * Upregulated DEGs (12): *ARG1, ARG2, HAMP, LRRC19, LTF, NOD1, OASL, PC, PRF1, PRLR, PTX3, ZDHHC8*
Developmental process	7.2	Downregulated DEGs (84): *ADA, ADAM19, ADAMTS12, ADAMTS15, ADAMTS2, ADIPOQ, AFF3, ANGPT1, ANPEP, ANXA2, ASB2, C1QTNF3, CA9, CCL11, CCR9, CD109, CDHR2, CDKN1A, CDX2, CHI3L1, CNTNAP1, COL12A1, COL1A1, COL1A2, COL3A1, COL5A1, COL5A2, CRISPLD2, DAPL1, EFEMP1, ELN, EPCAM, FKBP10, FUT1, GAL3ST1, GCNT3, GIP, GLUL, GSDME, INHBA, IRF4, ITGB4, KL, LGR5, LOX, LOXL2, MMP2, MYB, NANOS1, NFATC4, NKX2–3, NPR3, NPY1R, ONECUT1, P2RX7, PDX1, PKDCC, PPARGC1B, PSPH, RFLNB, RHOJ, RIPK3, RNF112, ROS1, RTN4RL1, SEMA3E, SERPINH1, SGCD, SLC27A4, SLC7A11, SLC9A1, SRC, SULF2, TGFA, TGFB3, TGM2, TMEM176B, TNC, TPPP3, VDR, VEGFC, VIL1, WNT5A, ZNF385A* Upregulated DEGs (38): *ANGPTL4, ARG2, ATF3, ATP6V1B1, BARX2, BHLHA15, CCND1, CDK1, CFTR, CLCF1, CYP1A2, DRGX, DYRK3, ECE2, ECT2, EOMES, GGT1, HSPA2, INSIG1, JPH1, LTF, MEIS1, MFSD2A, MLLT3, NEK2, PCDH19, PRLR, RFX2, SIK1, SLC1A2, SOX9, SP5, SPOCK1, TANC2, TGFB2, TPBG, ZBTB16, ZCWPW1*
Multicellular organismal process	7.0	Downregulated DEGs (60): *ADA, ADAMTS12, ADIPOQ, AHSG, ANGPT1, ASAH2, ASB2, CCL11, CD109, CDX2, CEL, CEMIP, CLDN4, CNTNAP1, COL1A1, COL3A1, CUX2, EDN3, EVC, FKBP10, FMO2, GCLC, GCNT3, GIP, HTR1B, INHBA, JCHAIN, LGR5, LYZ2, LYZ3, MMP2, MMRN1, NFATC4, NOD2, NPR3, NPY1R, OTOG, OTOGL, P2RX7, PI3, PKDCC, PNLIP, PPARGC1B, PROCR, PSPH, SEMA3E, SLC1A4, SLC27A4, SLC5A1, SLC7A11, SRC, SULF2, TGFB3, TGM2, TMPRSS15, TUSC3, VDR, VIL1, WNT5A, ZNF385A* Upregulated DEGs (21): *ARG2, ATP6V0A4, ATP6V1B1, CFTR, CYP1A2, DRGX, GRID1, HAMP, LTF, MEIS1, MFSD2A, MLLT3, PRLR, PRSS1, SLC1A2, SOX9, TANC2, TGFB2, TPBG, ZBTB16, ZDHHC8*
Metabolic process	6.6	Downregulated DEGs (133): *See Supplementary file Table S3C* Upregulated DEGs (72): *See Supplementary file Table S3D*
Biomineralisation	5.3	Downregulated DEGs (6): *ADA, ANXA2, COL1A1, COL1A2, LOX, PKDCC* Upregulated DEGs (1): *SOX9*
Biological regulation	4.7	Downregulated DEGs (195): *See Supplementary file Table S3E*. Upregulated DEGs (114): See Supplementary file Table S3F.
Localisation	4.7	Downregulated DEGs (76): *See Supplementary file Table S3G*. Upregulated DEGs (36): *See Supplementary file Table S3H.*

**Table 3:** Significant pathways identified by KEGG pathway enrichment associated with differentially expressed hepatic genes in MP4–7 (*n* = 40) compared with PP cows (*n* = 41).

Pathways	Enrichment FDR *p*-Value	Number of DEGs
Protein digestion and absorption	2.85 × 10^−6^	19
Metabolic pathways	1.95 × 10^−4^	82
Pancreatic secretion	6.74 × 10^−4^	13
Glutathione metabolism	3.38 × 10^−3^	9
Vitamin digestion and absorption	4.25 × 10^−3^	6
Amoebiasis	7.01 × 10^−3^	12
Arachidonic acid metabolism	7.01 × 10^−3^	10
Prostate cancer	1.60 × 10^−2^	10
Oestrogen signalling pathway	1.85 × 10^−2^	12
AGE-RAGE signalling pathway in diabetic complications	1.85 × 10^−2^	10
Ether lipid metabolism	1.85 × 10^−2^	7
Rheumatoid arthritis	2.00 × 10^−2^	10
ECM–receptor interaction	2.00 × 10^−2^	9
Relaxin signalling pathway	2.59 × 10^−2^	11
Viral protein interaction with cytokine and cytokine receptor	2.59 × 10^−2^	9
Taurine and hypotaurine metabolism	2.59 × 10^−2^	4
Arginine and proline metabolism	3.28 × 10^−2^	6
MAPK signalling pathway	3.43 × 10^−2^	18
Proteoglycans in cancer	3.43 × 10^−2^	14
Glycerolipid metabolism	3.43 × 10^−2^	7
Fat digestion and absorption	3.43 × 10^−2^	6
AMPK signalling pathway	3.56 × 10^−2^	10
Biosynthesis of amino acids	4.64 × 10^−2^	7
PI3K-Akt signalling pathway	4.91 × 10^−2^	21
Linoleic acid metabolism	4.91 × 10^−2^	5

**Table 4:** Summary of GO enrichment main functions of DEGs in the comparison between MP2–3 (*n* = 87) and PP cows (*n* = 41) in early lactation.

Functions	Enrichment Score	DEGs in the Function
Biomineralisation	8.6	Downregulated DEGs (7): *ADA, ANKH, COL1A1, COL1A2, LOX, PKDCC, SLC24A3* Upregulated DEGs (1): *SPP1*
Interspecies interaction between organisms	7.8	Downregulated DEGs (26): *ADIPOQ, ANPEP, CASP1, CCDC80, CHGA, gzmA, IL22RA1, IRF4, JCHAIN, LCN2, LEAP2, LYPD8, MST1R, NLRP1, NLRP6, NOD2, PLA2G1B, PNLIPRP2, POU2AF1, PRKCD, PYCARD, REG4, RIPK3, SARM1, SLC7A1, VIL1* Upregulated DEGs (5): *ARG1, FER1L6, LTF, PRLR, VNN1*
Multicellular organismal process	6.8	Downregulated DEGs (55): *ADA, ADAMTS12, ADIPOQ, AHSG, ANGPT1, ANKS6, AQP3, ASAH2, CCL11, CCND2, CDX2, CEL, CELA1, CLDN4, COL1A1, COL3A1, CUX2, FABP2, FOXS1, GCLC, GIP, HTR1B, INHBA, IQCB1, JCHAIN, LYZ2, LYZ3, MYO1A, NFATC4, NOD2, NPR3, PI3, PIGR, PKDCC, PNLIP, PPARGC1B, PROCR, PSPH, SEMA3E, SGPL1, SLC1A4, SLC5A1, SLC7A11, SRC, STK39, TGFB3, TGM2, TIFAB, TMEM79, TMPRSS15, TUSC3, VDR, VIL1, VLDLR, WNT5A* Upregulated DEGs (9): *ATP6V1B1, CFTR, CYP1A2, FGF12, LTF, MFSD2A, PRLR, SPP1, TPBG*
Developmental process	6.6	Downregulated DEGs (79)*: See Supplementary file Table S5B*. Upregulated DEGs (16)*: See Supplementary file Table S5C.*
Biological regulation	6.3	Downregulated DEGs (192)*: See Supplementary file Table S5D*. Upregulated DEGs (52)*: See Supplementary file Table S5E.*
Localisation	5.1	Downregulated DEGs (77)*: See Supplementary file Table S5F*. Upregulated DEGs (12)*: See Supplementary file Table S5G*.
Immune system process	4.7	Downregulated DEGs (28)*: ADA, CCL26, CCR9, CDH17, CHGA, CTSL, DPEP1, ENPP3, FRK, IRAK3, IRF4, JCHAIN, LOC100139916, LOC504295, MCOLN2, NLRP6, NOD2, PLA2G1B, POU2AF1, PRKCD, PSMB8, PSMB9, PYCARD, RFTN1, RIPK3, SARM1, SRC, STK39* Upregulated DEGs (6)*: CD70, CLCF1, LTF, NLRP1, TNFSF9, VNN1*
Locomotion	4.4	Downregulated DEGs (10)*: ANGPT1, ARHGEF16, CCL26, CHGA, DEFB13, DPEP1, LOX, PRKCD, VEGFC, WNT5A* Upregulated DEGs (1)*: TPBG*
Response to stimulus	4.1	Downregulated DEGs (91)*: See Supplementary file Table S5H*. Upregulated DEGs (21)*: See Supplementary file Table S5I*.

**Table 5:** Significant pathways identified by KEGG pathway enrichment associated with differentially expressed hepatic genes in MP2–3 (*n* = 87) compared with PP cows (*n* = 41).

Pathways	Enrichment FDR *p*-Value	Number of DEGs
Protein digestion and absorption	4.19 × 10^−4^	14
Pancreatic secretion	4.19 × 10^−4^	12
Metabolic pathways	1.09 × 10^−3^	66
Relaxin signalling pathway	1.25 × 10^−2^	11
AMPK signalling pathway	2.15 × 10^−2^	10
Glycerolipid metabolism	2.15 × 10^−2^	7
Fat digestion and absorption	2.15 × 10^−2^	6
Cytokine–cytokine receptor interaction	2.43 × 10^−2^	18
Ether lipid metabolism	2.43 × 10^−2^	6
Oestrogen signalling pathway	2.49 × 10^−2^	10
Viral protein interaction with cytokine and cytokine receptor	2.49 × 10^−2^	8
Prostate cancer	2.59 × 10^−2^	8
Linoleic acid metabolism	2.61 × 10^−2^	5
PI3K-Akt signalling pathway	2.75 × 10^−2^	19
Metabolism of xenobiotics by cytochrome P450	3.79 × 10^−2^	6

**Table 6:** Summary of GO enrichment main functions of DEGs in the comparison between MP4–7 (*n* = 40) and MP2–3 cows (*n* = 87) in early lactation.

Functions	Enrichment Score	DEGs in the Function
Immune system process	7.4	Downregulated DEGs (3)*: AGER, LGALS2, MCOLN2* Upregulated DEGs (8): *BCL6, CASP4, CD247, IFI16, LBP, NOD1, PTX3, TRAT1*
Biological regulation	4.4	Downregulated DEGs (9)*: AGER, IGF2BP3, LGALS2, LGR5, MCOLN2, NFATC4, NKD1, WFDC2, ZFPM1* Upregulated DEGs (34): *ATF3, BCL6, BHLHA15, CASP4, CCL24, CCL5, CCL8, CCNL1, CD247, CDKN2A, CHAC1, CHRM1, DNAJB1, FBXW5, HOPX, HSPA1A, IFI16, LBP, MECOM, MICAL2, MYOM1, NOD1, PTX3, RAB20, RELL1, RFX2, S100A1, SLCO4A1, SMAP2, SOCS3, SPIDR, STEAP4, TAGLN3, TRAT1*
Cellular process	3.5	Downregulated DEGs (10)*: AGER, DNAAF3, DNER, LGALS2, LGR5, MCOLN2, NFATC4, NKD1, SLC15A1, ZFPM1* Upregulated DEGs (38): *AEN, ATF3, ATP6V0D2, ATP6V1C2, BCL6, BHLHA15, CASP4, CCL24, CCL5, CCL8, CCNL1, CD247, CDKN2A, CHAC1, CHRM1, DNAJB1, FBXW5, HOPX, HSPA1A, HSPA6, HSPH1, KRBA2, LBP, MICAL2, MYOM1, NOD1, PTX3, RAB20, RFX2, SLC13A5, SLC5A8, SLCO4A1, SOCS3, SPIDR, SRM, STEAP4, TBATA, UCHL1*
Interspecies interaction between organisms	3.2	Downregulated DEGs (0)*:* Upregulated DEGs (6): *CASP4, CCL8, FKBP5, LBP, NOD1, PTX3*
Response to stimulus	3.1	Downregulated DEGs (2)*: AGER, NFATC4* Upregulated DEGs (19): *AEN, ATF3, BCL6, BHLHA15, CASP4, CHAC1, CHRM1, DNAJB1, FKBP5, HSPA6, LBP, NOD1, PTX3, RAB20, SLC13A5, SOCS3, SPIDR, SRM, TRAT1*

**Table 7:** Significant pathways identified by KEGG pathway enrichment associated with differentially expressed hepatic genes in MP4–7 (*n* = 40) compared with MP2–3 cows (*n* = 87).

Pathways	Enrichment FDR *p*-Value	Number of DEGs
Lipid and atherosclerosis	3.43 × 10^−3^	5
Protein processing in endoplasmic reticulum	3.43 × 10^−3^	4
Collecting duct acid secretion	3.43 × 10^−3^	2
Viral protein interaction with cytokine and cytokine receptor	3.43 × 10^−3^	3
NOD-like receptor signalling pathway	3.43 × 10^−3^	4
Rheumatoid arthritis	3.43 × 10^−3^	3
Oestrogen signalling pathway	5.93 × 10^−3^	3
Legionellosis	5.93 × 10^−3^	2
Glutathione metabolism	5.93 × 10^−3^	2
Longevity regulating pathway—multiple species	5.93 × 10^−3^	2
Wnt signalling pathway	6.41 × 10^−3^	3
Chronic myeloid leukaemia	6.41 × 10^−3^	2
Influenza A	6.41 × 10^−3^	3
Synaptic vesicle cycle	6.41 × 10^−3^	2
Chemokine signalling pathway	6.41 × 10^−3^	3
Antigen processing and presentation	6.88 × 10^−3^	2

## Data Availability

The RNA-seq FASTQ data can be obtained from the European Nucleotide Archive at https://www.ebi.ac.uk/ena/browser/home (accessed on 1 March 2023) with the accession number of ERP124149, ERP125646.

## Supplementary Materials

The supporting information can be downloaded at https://www.mdpi.com/article/10.3390/ijms24129906/s1.
