# Supplementary material for: Hepatic Global Transcriptomic Profiles of Holstein Cows According to Parity Reveal Age-Related Changes in Early Lactation
Source: Int J Mol Sci. 2023 Jun 8;24(12):9906. doi: 10.3390/ijms24129906 (PMC10298156; doi:10.3390/ijms24129906)
Supplement: Supplementary file 1 [file ijms-24-09906-s001.zip › Figure S1.docx]

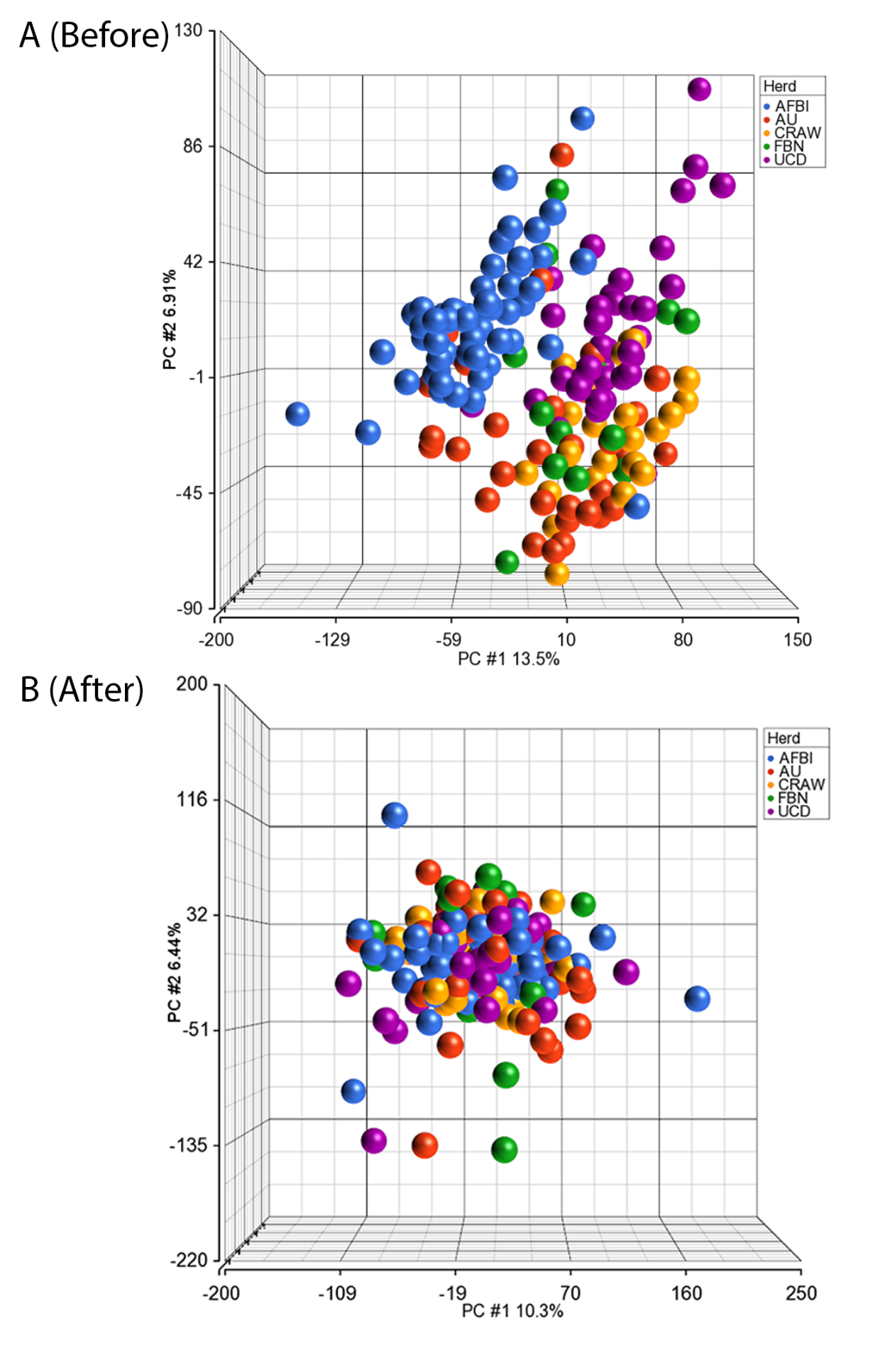


**Supplementary file Figure S1**. Principal component analysis for the normalised read counts (RPKM) before (A) and after (B) applying herd effect control. AFBI: Agri-Food and Biosciences Institute Hillsborough, Northern Ireland; AU: Aarhus University, Denmark; CRA: Walloon Agricultural Centre, Belgium; FBN: Leibniz Institute for Farm Animal Biology, Germany; UCD: University College Dublin, Ireland. Each sphere represents one cow. After the herd control the expression patterns were no longer separated by herd.
